# Comprehensive analysis of gene expression patterns in Friedreich's ataxia fibroblasts by RNA sequencing reveals altered levels of protein synthesis factors and solute carriers

**DOI:** 10.1242/dmm.030536

**Published:** 2017-11-01

**Authors:** Jill Sergesketter Napierala, Yanjie Li, Yue Lu, Kevin Lin, Lauren A. Hauser, David R. Lynch, Marek Napierala

**Affiliations:** 1 University of Alabama at Birmingham, Department of Biochemistry and Molecular Genetics, UAB Stem Cell Institute, 1825 University Blvd., Birmingham, Alabama 35294, USA; 2 University of Texas MD Anderson Cancer Center, Department of Molecular Carcinogenesis, Center for Cancer Epigenetics, Science Park, Smithville, Texas 78957, USA; 3 Departments of Neurology and Pediatrics, Children's Hospital of Philadelphia, Abramson Research Center Room 502, Philadelphia, PA 19104, USA; 4 Department of Molecular Biomedicine, Institute of Bioorganic Chemistry, Polish Academy of Sciences, Poznan, 61-704, Poland

**Keywords:** Friedreich's ataxia, Fibroblasts, Translation, Solute carriers, RNA sequencing

## Abstract

Friedreich's ataxia (FRDA) is an autosomal recessive neurodegenerative disease usually caused by large homozygous expansions of GAA repeat sequences in intron 1 of the frataxin (*FXN*) gene. FRDA patients homozygous for GAA expansions have low *FXN* mRNA and protein levels when compared with heterozygous carriers or healthy controls. Frataxin is a mitochondrial protein involved in iron–sulfur cluster synthesis, and many FRDA phenotypes result from deficiencies in cellular metabolism due to lowered expression of *FXN*. Presently, there is no effective treatment for FRDA, and biomarkers to measure therapeutic trial outcomes and/or to gauge disease progression are lacking. Peripheral tissues, including blood cells, buccal cells and skin fibroblasts, can readily be isolated from FRDA patients and used to define molecular hallmarks of disease pathogenesis. For instance, *FXN* mRNA and protein levels as well as *FXN* GAA-repeat tract lengths are routinely determined using all of these cell types. However, because these tissues are not directly involved in disease pathogenesis, their relevance as models of the molecular aspects of the disease is yet to be decided. Herein, we conducted unbiased RNA sequencing to profile the transcriptomes of fibroblast cell lines derived from 18 FRDA patients and 17 unaffected control individuals. Bioinformatic analyses revealed significantly upregulated expression of genes encoding plasma membrane solute carrier proteins in FRDA fibroblasts. Conversely, the expression of genes encoding accessory factors and enzymes involved in cytoplasmic and mitochondrial protein synthesis was consistently decreased in FRDA fibroblasts. Finally, comparison of genes differentially expressed in FRDA fibroblasts to three previously published gene expression signatures defined for FRDA blood cells showed substantial overlap between the independent datasets, including correspondingly deficient expression of antioxidant defense genes. Together, these results indicate that gene expression profiling of cells derived from peripheral tissues can, in fact, consistently reveal novel molecular pathways of the disease. When performed on statistically meaningful sample group sizes, unbiased global profiling analyses utilizing peripheral tissues are critical for the discovery and validation of FRDA disease biomarkers.

## Abstract (precis)

**Summary:** Transcriptome profiling of Friedreich's ataxia fibroblasts by RNA sequencing reveals that this peripheral tissue can be used as a disease model for gene expression biomarker discovery.

## INTRODUCTION

Friedreich's ataxia (also known as FRDA or FA; OMIM229300) is the most prevalent inherited ataxia in humans, with a population frequency of 1-2:50,000 (Campuzano et al., 1996), and is caused by deficient levels of the mitochondrial protein frataxin (encoded by *FXN*) (Campuzano et al., 1997). The majority of FRDA patients are homozygous for large expansions of GAA repeat sequences in intron 1 of the *FXN* gene, whereas a small fraction of patients are compound heterozygotes with an expanded GAA repeat sequence in one *FXN* allele and a missense or nonsense mutation in the other (Cossée et al., 1999). Both types of lesions result in reduced levels of *FXN* mRNA and protein when compared with heterozygous carriers and healthy controls.

An inverse correlation exists between frataxin protein level and disease severity (Evans-Galea et al., 2012). Characteristic symptoms of FRDA include discoordination, slurred speech, muscle weakness, sensory loss and cardiomyopathy (www.omim.org). In addition, optic atrophy (Porter et al., 2007), auditory defects (Rance et al., 2010) and scoliosis (reviewed in Delatycki et al., 2000) are also observed in some patients. There exists great clinical heterogeneity between FRDA patients in timing of disease onset, progression rate, and manifestation of particular symptoms. Although the age of disease onset has been repeatedly linked with the length of the shorter of the two expanded GAA repeats in *FXN* (GAA1) (Campuzano et al., 1997; Deutsch et al., 2010; Lazaropoulos et al., 2015), additional molecular markers that correlate with disease features are lacking in the field.

Transcriptome signatures associated with specific disease states can provide great information about pathogenic mechanisms and bring to light priority gene expression biomarker candidates. Screening methods such as microarrays and bead arrays have been used to define disease signatures, including FRDA lymphoblast signatures (Coppola et al., 2011; Haugen et al., 2010). The current progress in next-generation sequencing allows us to conduct expression profiling with much greater sensitivity and accuracy. This approach has not been employed for large-scale analyses in FRDA research.

FRDA pathology is most commonly observed in neuronal and cardiac tissue, but these affected tissues are not attainable from living patients. Most human specimens used for mechanistic studies of FRDA are provided by patients who generously donate peripheral tissue samples, such as blood, buccal swabs and skin punch biopsies, for research purposes. Although not directly involved in FRDA pathogenesis, peripheral tissues from patients are deficient of frataxin and can serve as models to collect data associated with stable molecular features of the disease. One advantage of fibroblast cells is that their physiology is unlikely to be perturbed by acute changes in daily living activities, such as fluctuations in diet or environment, making them a suitable model for defining persistent gene expression patterns of FRDA.

We routinely establish primary fibroblast cell lines from skin biopsies and currently have banked more than 50 unique lines from FRDA patient donors (Li et al., 2016). Importantly, the FRDA patients associated with these samples exhibit a wide range of molecular and clinical characteristics, allowing for selection of a cohort that represents a diverse FRDA sampling pool. Herein, we generated transcriptome profiles for 18 FRDA and 17 unaffected [control (CTRL)] fibroblast lines. We performed gene ontology (GO) analyses and compared previously published FRDA blood gene expression signatures with the significantly changed genes in fibroblasts, and discovered common dysregulation of several pathways as well as specific genes. Our results indicate that gene expression patterns specific to FRDA can emerge from distinct peripheral tissue types.

## RESULTS

### RNA sequencing identifies differentially expressed genes in CTRL and FRDA primary fibroblasts

In order to identify gene expression patterns specific for FRDA, we performed transcriptome profiling using an ultra-deep unbiased RNA sequencing (RNA-Seq) approach. The RNA samples analyzed were prepared from 16 primary fibroblast FRDA cell lines and two unaffected individual (CTRL) cell lines currently held in our repository (Li et al., 2016), along with 15 primary CTRL fibroblast cell lines and two FRDA lines acquired from Coriell Cell Repositories (Table S1). The FRDA and CTRL cohorts were matched for sex and age of sampling, and consisted of seven females and 11 males (FRDA) or nine females and eight males (CTRL), with a mean sampling age of 36.3 years (13-70 years; FRDA) or 30.1 years (1 day to 64 years; CTRL), as described in Li et al. (2015b). The patients who provided samples for the FRDA cohort began exhibiting disease symptoms anywhere from 4-41 years of age (mean age of onset: 17.7 years; Table S1), also described in Li et al. (2015b). In this FRDA cohort, the average lengths of the GAA tracts are 454 and 898 repeats for *FXN* alleles 1 and 2 (GAA1 and GAA2), respectively (Table S1), whereas the number of GAA triplets did not exceed 40 repeats in any of the CTRL fibroblast lines (Li et al., 2015b). We also performed statistical analyses to determine intra-group heterogeneity and found no significant variance between samples, nor were any outlier samples identified within either group (Fig. S1).

**Fig. 1.**
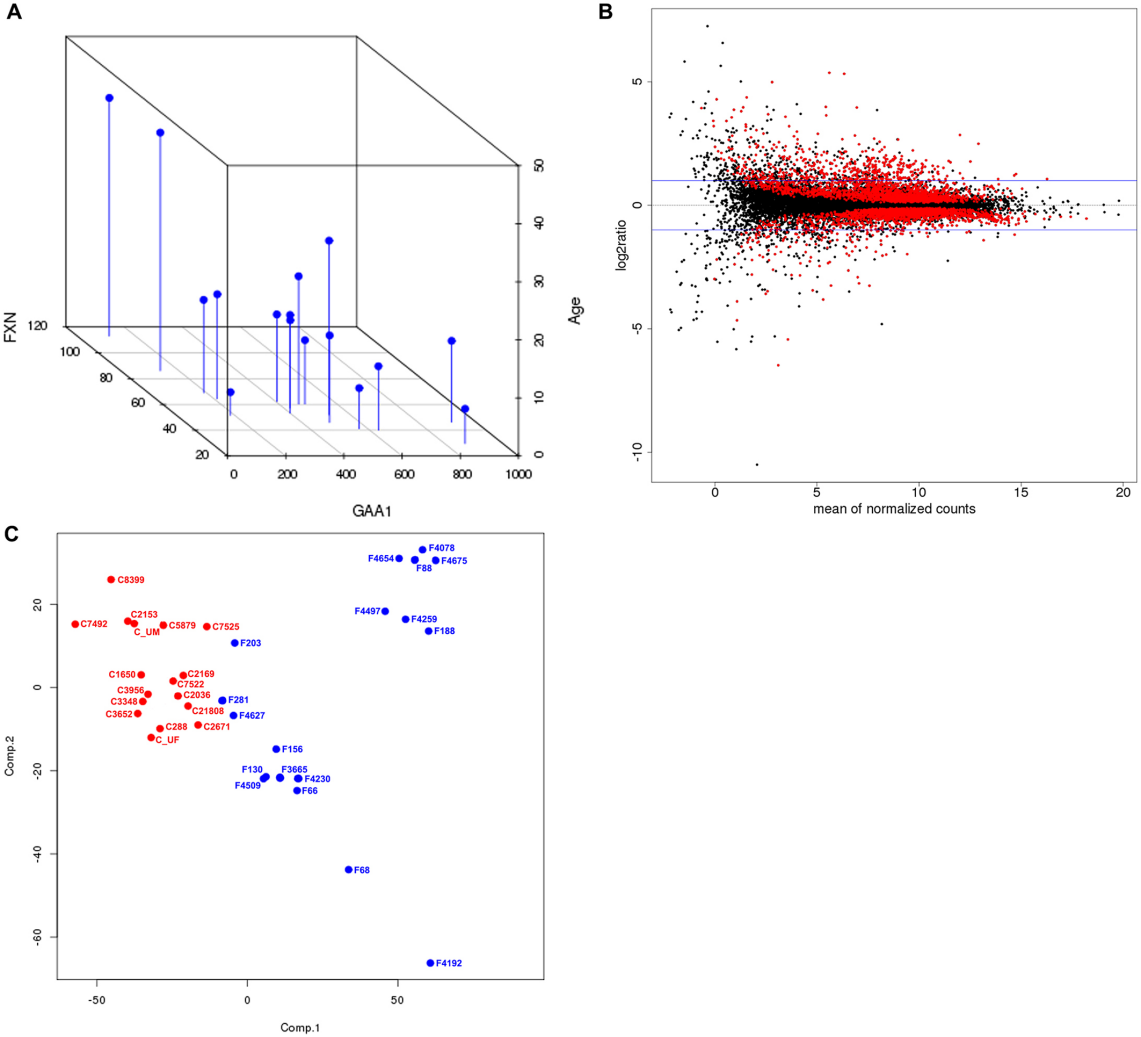
**Analysis of** RNA-Seq **data generated from fibroblast lines derived from 18 FRDA and 17 unaffected CTRL individuals.** (A) A three-dimensional plot of GAA1 repeat length on the *x*-axis (GAA1), age of disease onset on the *y*-axis (Age) and *FXN* expression by RNA-Seq on the *z*-axis (FXN) for each FRDA fibroblast sample is shown with a view angle of 140°. Pearson's correlation coefficients were determined for each comparison and are as follows: age versus GAA1: *R*=−0.49, *P*=0.05; age versus FXN: *R*=0.81, *P*=0.0001; GAA1 vs FXN: *R*=−0.76, *P*=0.0007. (B) The MA plot illustrates log_2_ ratio (*y*-axis) versus the mean normalized counts (*x*-axis) for transcripts detected by RNA-Seq. The red dots represent differentially expressed genes with FDR≤0.05 and the horizontal blue lines indicate cutoff points for two-fold changes in expression. (C) PCA of CTRL (C; red) and FRDA (F; blue) samples based on expression of significantly changed genes only.

RNAs isolated from all 35 fibroblast lines (18 FRDA and 17 CTRL) were reverse transcribed to generate sequencing libraries and sequenced by HiSeq2000 (Illumina). Approximately 27- to 39-million sequencing reads were generated for each fibroblast mRNA preparation and 82-93% of them were mapped on both ends to the human genome. In total, 16,531 transcripts were detected using a threshold of ≥10 tags in at least one sample. As previously published, *FXN* expression is significantly decreased ∼2.3-fold in FRDA samples relative to CTRL samples (*P*=4×10^−12^), albeit at variable levels (Table S1; Li et al., 2015b). Also, *FXN* expression correlates directly with the length of the shorter GAA tract (GAA1) and inversely with the age of disease onset (Fig. 1A), as previously described (Li et al., 2015b). Additional viewing angles of the three-dimensional plot are provided in Fig. S2.

Differential gene expression analysis between the FRDA and CTRL cohorts was conducted using the Bioconductor/R DESeq package (Anders and Huber, 2010). Expression levels of approximately 23% (3788) of all genes were significantly changed [false discovery rate (FDR)<0.05] between FRDA and CTRL groups (Fig. 1B, red dots) and ∼16% of these genes had a ≥two-fold change in expression (624 genes) (Fig. 1B). The list of 3788 genes is provided in Table S2. Principal component analysis (PCA) shows that the CTRL and FRDA groups are well separated based on the expression of the significantly changed genes (Fig. 1C).

**Fig. 2.**
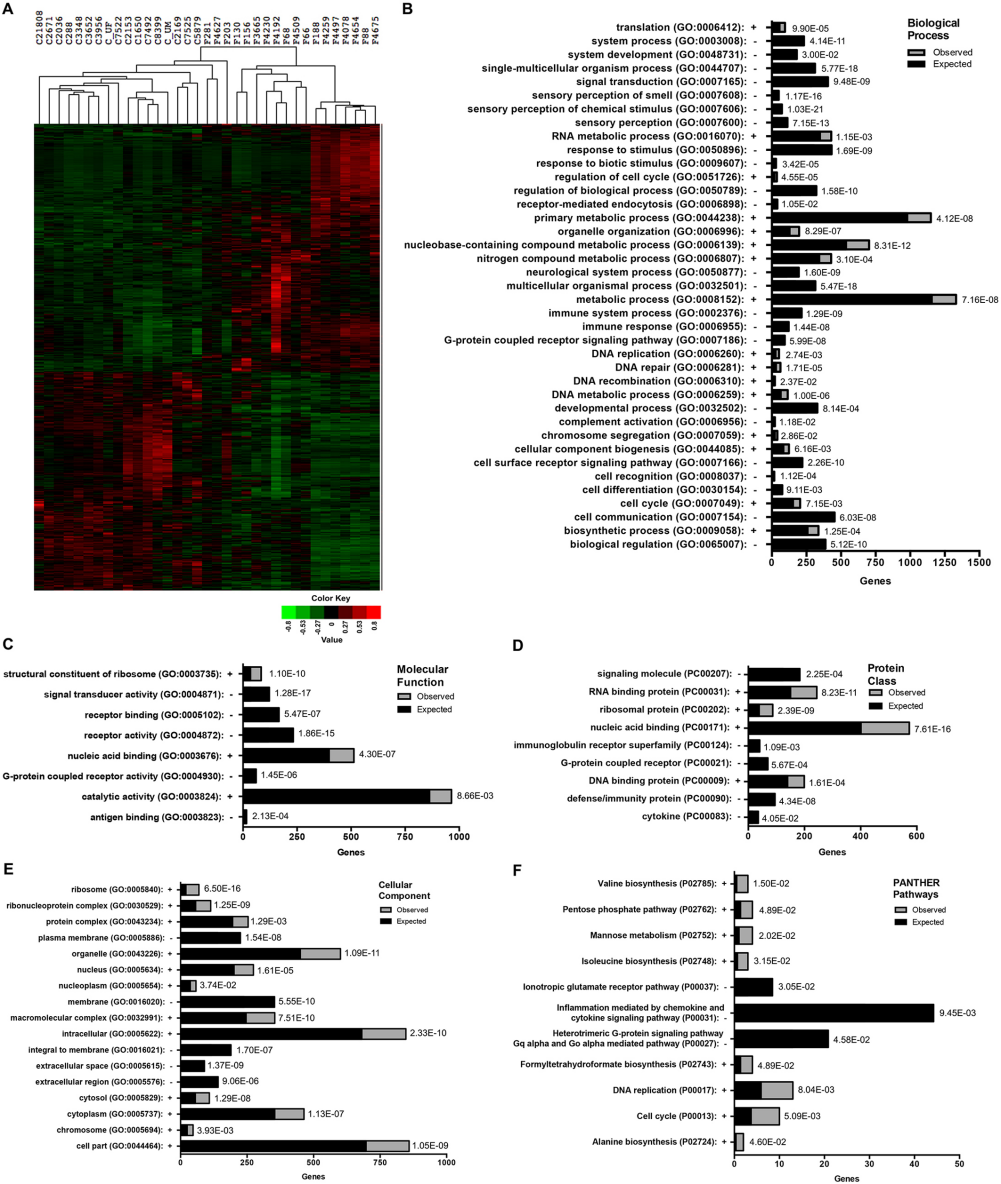
**GO analysis of significantly changed genes in FRDA fibroblasts.** (A) The CTRL (C) and FRDA (F) patient samples are organized by hierarchical clustering based on the normalized DESeq values for 3788 genes found to be significantly changed (FDR≤0.05) between the two groups. The expression levels of these genes are shown as a heatmap. Downregulated genes are shown in green and upregulated genes are shown in red, with the color intensity corresponding to the degree of change. (B-F) The PANTHER statistical over-representation test tool was used to determine over- and under-representation of defined GO classifications for the 3788 significantly changed genes in FRDA samples: (B) biological process; (C) molecular function; (D) protein class; (E) cellular component; (F) PANTHER pathways. Expected frequencies for each category are shown by black bars, whereas observed frequencies are indicated by gray shading. The over-representation (+) or under-representation (−) status is also indicated along each *y*-axis next to the GO term, which are listed alphabetically starting from the *x*-axis. The number of genes assigned to each category from the list of 3788 can be determined from the *x*-axis, and significance (*P*-value) for each category is given to the right of each bar.

Of the 3788 differentially expressed genes identified between the FRDA and CTRL cohorts (FDR<0.05), 2015 are upregulated (Fig. 2A, red) and 1773 are downregulated (Fig. 2A, green) in the FRDA cohort. Unsupervised hierarchical clustering of the CTRL and FRDA samples demonstrated a clear separation of the two groups, as indicated also by PCA (Figs 2A and 1C).

### GO and expression enrichment analyses define molecules and processes perturbed in FRDA fibroblasts

In order to determine which cellular processes, molecule classes or pathways are over- or under-represented in FRDA fibroblasts, the list of 3788 differentially expressed genes was used as the input for a statistical over-representation test within the protein analysis through evolutionary relationships (PANTHER, version 11.1) platform (Mi et al., 2013). This tool uses a binomial test to compare a user-provided gene list with a reference gene list (all genes in the PANTHER database for the selected organism) for each defined PANTHER GO term, protein class or pathway (Cho and Campbell, 2000; Mi et al., 2017). From our list, 3476 genes were mapped, whereas 312 were excluded from analysis as unmapped IDs. Bar plots are shown for major GO terms of ‘biological process’ (Fig. 2B), ‘molecular function’ (Fig. 2C), ‘protein class’ (Fig. 2D), ‘cellular component’ (Fig. 2E) and ‘PANTHER pathways’ (Fig. 2F). The GO-slim ontologies, as defined by PANTHER, were used for the biological process, molecular function and cellular component classifications (Mi et al., 2017, 2013, 2016).

The metabolic process category (GO:0008152) was over-represented and had the highest number of genes mapped for the GO term biological process (1331 genes observed/1156 genes expected; *P*=7.16E–08) (Fig. 2B). Genes mapping to categories pertaining to translation and RNA metabolic processes (GO:0006412, GO:0016070), as well as regulation of cell cycle (GO:0051726, GO:0007049) and nucleobase and nitrogen compound metabolic processes (GO:0006139, GO:0006807), were also observed in the differentially expressed gene list more than the expected frequency for those categories in the reference gene list, as indicated by the greater ratio of observed (Fig. 2B, gray)/expected (Fig. 2B, black) for each bar.

The over-representation of genes involved in translation and RNA metabolic processes carries over to the molecular function (GO:0003735), protein class (PC:00031, PC:00202) and cellular component (GO:0005840, GO:0030529) ontologies also, and primarily include categories relevant to ribosomal components and structure (Fig. 2C-E). Genes encoding proteins involved in valine, isoleucine and alanine biosynthesis (P02785, P02748 and P02724, respectively) are also over-represented in the differentially expressed genes list, as indicated by enrichment in these categories as classified by PANTHER pathways (Fig. 2F).

Genes mapping to categories pertaining to sensory perception (GO:0007606, GO:0007608 and GO: 000760) were among the most significantly under-represented, whereas the category metabolic processes involving nucleobase-containing compounds (GO:0006139) was the most significantly enriched (Fig. 2B). Several receptor-mediated signaling pathway categories also had fewer mapped genes than expected, as evident for nearly all GO terms: biological process (GO:0007165, GO:0007186, GO:0007166; Fig. 2B), molecular function (GO:0004871, GO:0005102, GO:0004872, GO:0004930; Fig. 2C), protein class (PC00207, PC00021, PC00083; Fig. 2D) and PANTHER pathways (P00037, P00031, P00027; Fig. 2F). Specifically, G-protein coupled-receptor signaling categories were consistently and significantly under-represented in our list of differentially expressed genes (Fig. 2B-D,F).

**Fig. 3.**
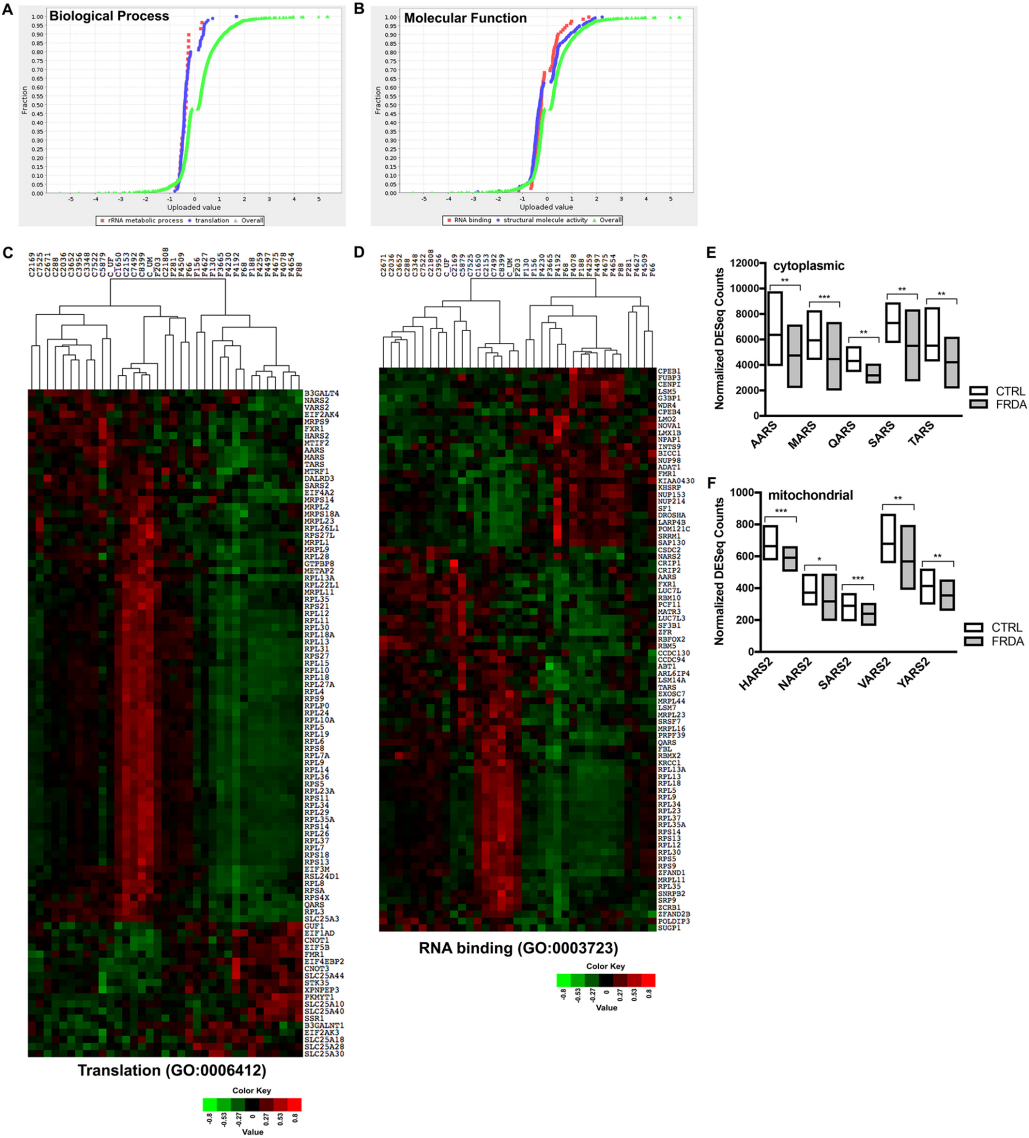
**Expression enrichment analysis reveals that genes required for protein synthesis are significantly under-expressed in FRDA fibroblasts.** (A,B) The list of 3788 significantly changed genes along with the calculated log_2_ ratio (FRDA/CTRL) for each gene was used as the input file for the PANTHER statistical enrichment test tool. The uploaded expression value (log_2_ ratio) for genes assigned to the plotted categories are indicated by the *x*-axis, whereas the fraction of genes assigned to each category is indicated by the *y*-axis. The distribution of expression values for all 3788 genes is given by the central green curve and shifts to the left or right indicate lower or higher expression levels, respectively, of the genes assigned to the plotted category. Each gene is indicated by a dot. Plots are shown for selected categories for the major GO terms (A) biological process and (B) molecular function. (C,D) The normalized DESeq counts were used to generate expression heatmaps for the significantly changed genes assigned to GO categories selected from enrichment analyses shown in A and B. The CTRL and FRDA fibroblast samples were arranged by unsupervised hierarchical clustering. Downregulated genes are shown in green and upregulated genes are shown in red, with the color intensity corresponding to the degree of change. (E,F) The normalized DESeq counts for the indicated cytoplasmic aaRS genes (E) or mitochondrial aaRS genes (F) are shown for the CTRL and FRDA sample groups. The central line in each bar denotes the mean, and the upper and lower limits denote the maximum and minimum expression values for the gene in each sample group. The level of statistical significance is indicated by asterisks as follows: ***P≤0.0001; **P≤0.001; *P≤0.01.

Next, the PANTHER statistical enrichment test tool was used to determine whether expression changes of genes assigned to particular ontology terms are distributed non-randomly throughout the overall list of expression values for the 3788 significantly changed genes between CTRL and FRDA samples. The PANTHER statistical enrichment test uses the Mann–Whitney *U*-test (Wilcoxon rank-sum test) to determine the significance of enriched or under-represented categories (Mi et al., 2013). The list of 3788 differentially expressed genes (FDR<0.05) along with their associated log_2_ ratio values served as the input for statistical enrichment tests. Results for significantly over- or under-represented categories for all GO terms, including gene counts and *P*-values, are shown in Table 1. Expression of genes mapped to the GO categories translation (GO:0006412; *P*=1.91E–11) and rRNA metabolic process (GO:0016072; *P*=1.11E–03) under the term of biological process is significantly downregulated in FRDA when compared to the overall distribution of gene expression values (log_2_ ratio; FRDA/CTRL) (Fig. 3A and Table 1). Likewise, under the molecular function term, the GO categories of RNA binding (GO:0003723; *P*=4.27E–02) and structural molecule activity (GO:0005198; *P*=6.20E–06) are significantly enriched for genes showing decreased expression in FRDA fibroblasts (Fig. 3B and Table 1). In order to visualize these gene expression changes for individual FRDA samples, heatmaps were generated using the normalized DESeq counts for genes included in the GO categories of ‘translation’ (Fig. 3C) and ‘RNA binding’ (Fig. 3D).

**Table 1.**
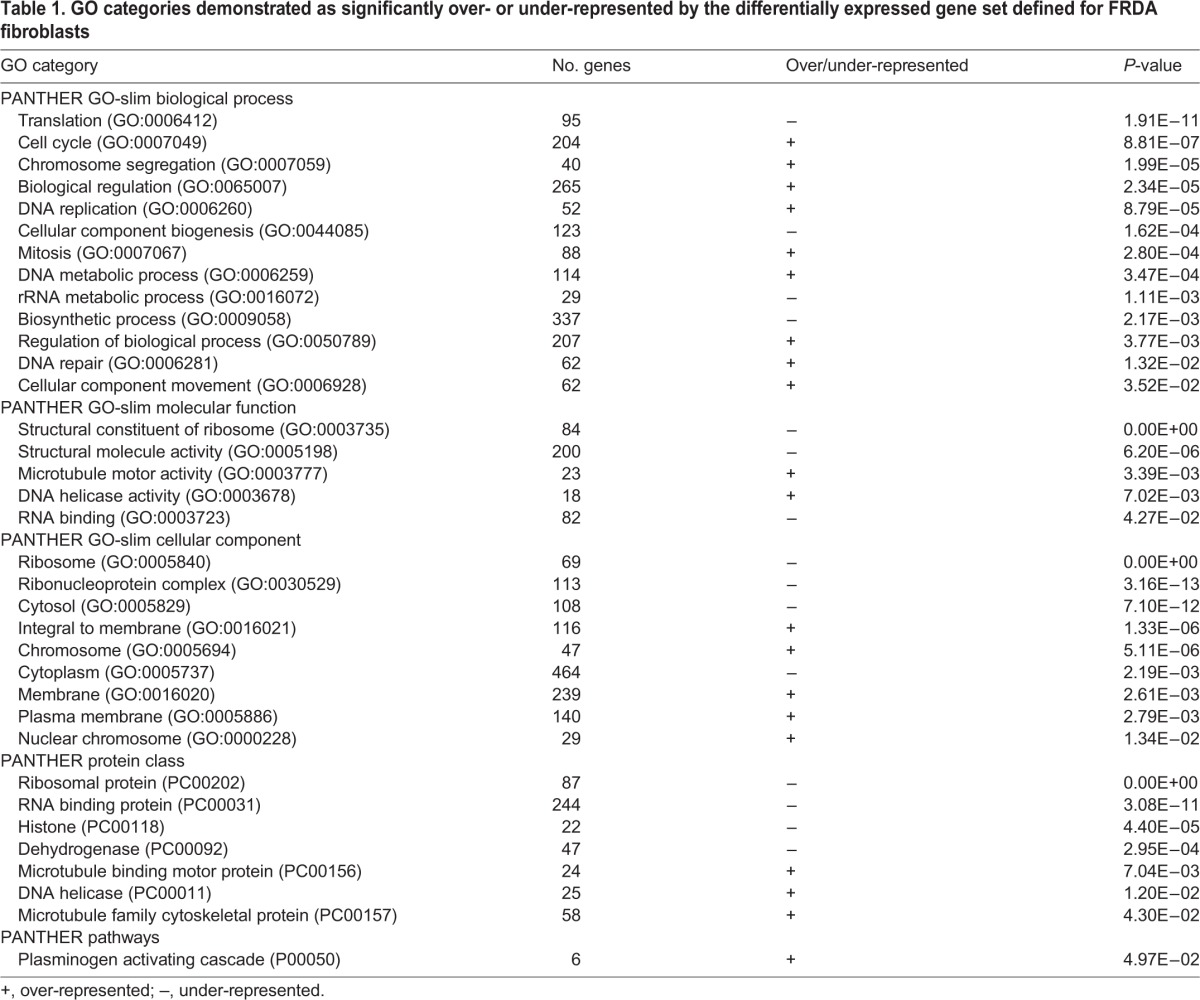
**GO categories demonstrated as significantly over- or under-represented by the differentially expressed gene set defined for FRDA fibroblasts**

Overall, expression levels of ribosomal protein L (*RPL*) and ribosomal protein S (*RPS*) genes, which encode 60S and 40S ribosomal proteins, respectively, are significantly downregulated in the majority of FRDA samples (Fig. 3C,D). Also, MRPL and MRPS family members, which are nuclear-encoded genes that produce mitochondrial 39S and 28S ribosomal proteins, respectively, are also significantly under-expressed in FRDA fibroblasts (Fig. 3C,D). Finally, we noticed that several genes encoding cytoplasmic aminoacyl-tRNA synthetases (aaRS) or mitochondrial aaRS enzymes (aaRS2) are significantly depleted in FRDA samples compared to CTRL samples (*AARS*, *MARS*, *QARS*, *TARS*, *HARS2*, *NARS2*, *SARS2*, *VARS2*) (Fig. 3C). Re-analysis of the list of 3788 differentially expressed genes revealed two additional aaRS genes, *SARS* and *YARS2*, that were not annotated to the translation GO category (GO:0006412). The normalized DESeq counts for the genes encoding cytoplasmic and mitochondrial aaRS enzymes are plotted for the CTRL and FRDA sample groups (Fig. 3E,F). Categories pertaining to protein synthesis under PANTHER protein class were also significantly under-represented in terms of gene expression in FRDA samples, including ribosomal protein (PC00202; *P*=0.00E00) and RNA binding protein (PC00031; *P*=3.08E–11) (Table 1). This further demonstrates an overall deficiency in expression of genes necessary for cytoplasmic and mitochondrial ribosomal biogenesis as well as general protein biosynthesis in FRDA fibroblasts.

**Fig. 4.**
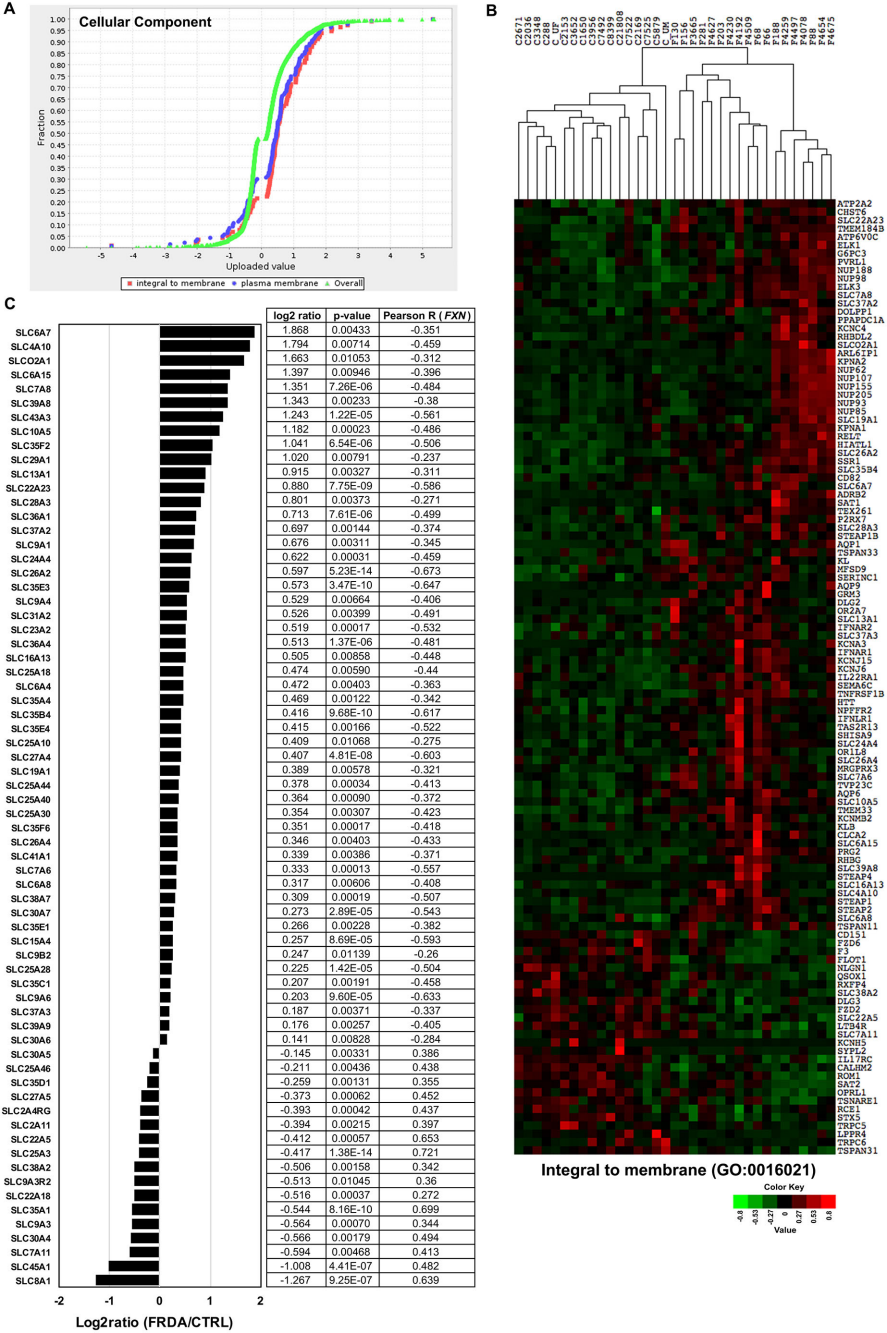
**Expression enrichment analysis reveals altered expression of genes encoding membrane solute carriers in FRDA fibroblasts.** (A) The list of 3788 significantly changed genes along with the calculated log_2_ ratio (FRDA/CTRL) for each was used as the input file for the PANTHER statistical enrichment test tool. The uploaded expression value (log_2_ ratio) for genes assigned to the plotted categories are indicated by the *x*-axis, whereas the fraction of genes assigned to each category is indicated by the *y*-axis. The distribution of expression values for all 3788 genes is given by the central green curve and shifts to the left or right indicate lower or higher expression levels, respectively, of the genes assigned to the plotted category. Each gene is indicated by a dot. Plots are shown for selected categories for the major GO term cellular component. (B) The normalized DESeq counts were used to generate expression heatmaps for the significantly changed genes assigned to the category ‘integral to membrane’ (GO:0016021). The CTRL and FRDA fibroblast samples were arranged by unsupervised hierarchical clustering. Downregulated genes are shown in green and upregulated genes are shown in red, with the color intensity corresponding to the degree of change. (C) The bar plot shows the expression [log_2_ ratio (FRDA/CTRL)] for all SLC family genes significantly changed in FRDA cells. The log_2_ ratio, *P*-value and Pearson correlation coefficient (*R*; determined for correlation with *FXN* expression across all 35 samples) are given for each gene to the right of the plot.

Conversely, the cellular component GO categories of integral to membrane (GO:0016021; *P*=1.33E–06) and plasma membrane (GO:0005886; *P*=2.79E–03) are significantly enriched for genes upregulated in FRDA fibroblasts (Fig. 4A). A heatmap was generated to visualize expression changes for genes mapped to the category ‘integral to membrane’ for each CTRL and FRDA sample (Fig. 4B). The expression of 68 solute carrier (SLC) family genes and SLC regulators is dysregulated in the majority of FRDA samples, with 75% being upregulated (Figs 3C and 4B,C). In particular, we noticed that the expression levels of eight mitochondrial SLC family 25 genes (SLC25) are upregulated in the FRDA group (Figs 3C and 4C). The SLC25 proteins are localized to the inner mitochondrial membrane and are responsible for transporting various metabolites, such as ATP/ADP, amino acids, iron, malate, ornithine and citrulline, into and out of the matrix (Haitina et al., 2006; Palmieri, 2004). In fact, the most significantly changed SLC gene in FRDA cells is *SLC25A3* (log_2_ ratio=−0.41693; *P*=1.38E–14), which codes for the mitochondrial phosphate carrier protein responsible for phosphate uptake into the matrix at the final stage of oxidative phosphorylation (Jabs et al., 1994). Also, the expression of *SLC25A3* is highly correlated with *FXN* expression across all 35 samples [Pearson correlation coefficient (*R*)=0.721]. The largest changes in expression between FRDA and CTRL samples occur for plasma membrane transporters usually expressed in CNS tissues (*SLC6A7*, log_2_ ratio=1.86812; *P*=0.00432) and cardiac cells (*SLC8A1*, log_2_ ratio=−1.26674; *P*=9.25E–07) (Fig. 4B,C). Taken together, the consistent dysregulation of numerous SLC family members suggests that flux of small molecules between cellular compartments and/or between cells might be altered in FRDA, and suggest these transporters as novel potential therapeutic targets.

Under the PANTHER protein class ontology, a significant enrichment in upregulated genes is observed for the categories microtubule family cytoskeletal protein (PC00157; *P*=4.30E–02) and microtubule binding motor protein (PC00156; *P*=7.04E–03) for FRDA samples (Table 1, Fig. S3A). Also, because several categories associated with DNA metabolic processes (GO:0006259; *P*=3.47E–04) were significantly enriched in FRDA samples (Table 1), genes mapped to the terms DNA replication (GO:0006260; *P*=8.79E–05) and DNA repair (GO:0006281; *P*=1.32E–02) were also plotted relative to the overall distribution and the expression values for these genes (Fig. S3B). Accompanying heatmaps illustrate the expression values for genes in these categories in all fibroblast samples (Fig. S3C-F). Visualization of the expression data by individual samples revealed that a subset of seven FRDA cell lines had markedly high expression of large groups of genes in each category (Fig. S3C-F). Based on molecular, clinical and demographic data currently available for these patients, there are no overt distinctions between this subset and the remaining 11 patients in the FRDA cohort (Table S1, Fig. S1). Therefore, attention was focused on analyzing differentially expressed genes that showed uniform changes across each cohort.

Several kinesin family member genes showed uniform upregulation across the FRDA cohort, including *KIF16B* and *KIF21A* (Fig. S3C,E). Moreover, upregulation of the axonal transport gene *KIF1B* is observed, consistent with previously published data demonstrating upregulation of *Kif1b* in dorsal root ganglia (DRG) neurons of the YG8R FRDA humanized mouse model (Shan et al., 2013). The *GABARAP* gene encodes a γ-aminobutyric-acid-A-receptor-associated protein that coordinates interaction with the cytoskeleton to cluster neurotransmitter receptors (Wang et al., 1999), and is consistently downregulated among the FRDA samples (Fig. S3C). Also showing decreased expression in FRDA fibroblasts is the *KATNA1* (katanin p60 subunit A1) gene, which encodes an enzyme that severs microtubules in neurons, a function that is critical for axon dynamics (Karabay et al., 2004).

As previously reported, a number of genes involved in DNA replication and repair are more under-expressed in FRDA cells relative to CTRL cells (Fig. S3D,F) (Bhalla et al., 2016; Coppola et al., 2011; Haugen et al., 2010; Martelli et al., 2007). Included in the list of downregulated genes are those encoding the nuclear and mitochondrial DNA glycosylases [*NTHL1* (nth like DNA glycosylase 1), *P*=5.31E–17; *MPG* (N-methylpurine DNA glycosylase), *P*=0.0001; *OGG1* (8-oxoguanine DNA glycosylase), *P*=0.004], DNA endonucleases [*ERCC1* (ERCC excision repair 1, endonuclease non-catalytic subunit), *P*=0.001; *ERCC3* (ERCC excision repair 3, TFIIH core complex helicase subunit), *P*=0.008], and the DNA damage and replication stress checkpoint protein RAD17 [*RAD17* (*RAD17* checkpoint clamp loader component), *P*=0.006]. Decreased gene expression for factors involved in all major DNA repair pathways is noted in FRDA cells, including double-strand break repair [*NBN* (nibrin), *P*=0.0001], mismatch repair [*PMS1* (PMS1 homolog 1, mismatch repair system component), *P*=0.001; *PMS2* (PMS1 homolog 2, mismatch repair system component), *P*=0.009] and nucleotide excision repair [*RAD23B* (RAD23 homolog B, nucleotide excision repair protein), *P*=0.0003; *GTF2H4* (general transcription factor IIH subunit 4), *P*=0.009]. Moreover, a nuclear-encoded gene encoding a mitochondrial topoisomerase [*TOP1MT* (DNA topoisomerase I, mitochondrial), *P*=0.002] is downregulated, suggesting a potential strain on mitochondrial DNA replication in FRDA cells.

The Reactome Pathway database (Fabregat et al., 2016; Mi et al., 2017) was also selected as an output measure for statistical enrichment (Table 2). In agreement with previous GO analyses, the most significantly enriched pathways include cell cycle regulation (R-HSA-1640170) and RHO GTPase signaling (R-HSA-194315). Conversely, pathways pertaining to general metabolism (R-HSA-1430728), gene expression (R-HSA-74160) and translation (R-HSA-72766) are among the most significantly under-represented in FRDA fibroblast cells in terms of expression levels of their comprised genes (Table 2). Additionally, the 3788 significantly differentially expressed genes (FDR<0.05) were used as an input list for the Enrichr platform (Chen et al., 2013; Kuleshov et al., 2016). The results indicate that the top enriched pathways shown by the Reactome Pathway database are also among the most significantly represented when the KEGG 2016 and WikiPathways 2016 databases are cross-examined (Table S3).

**Table 2.**
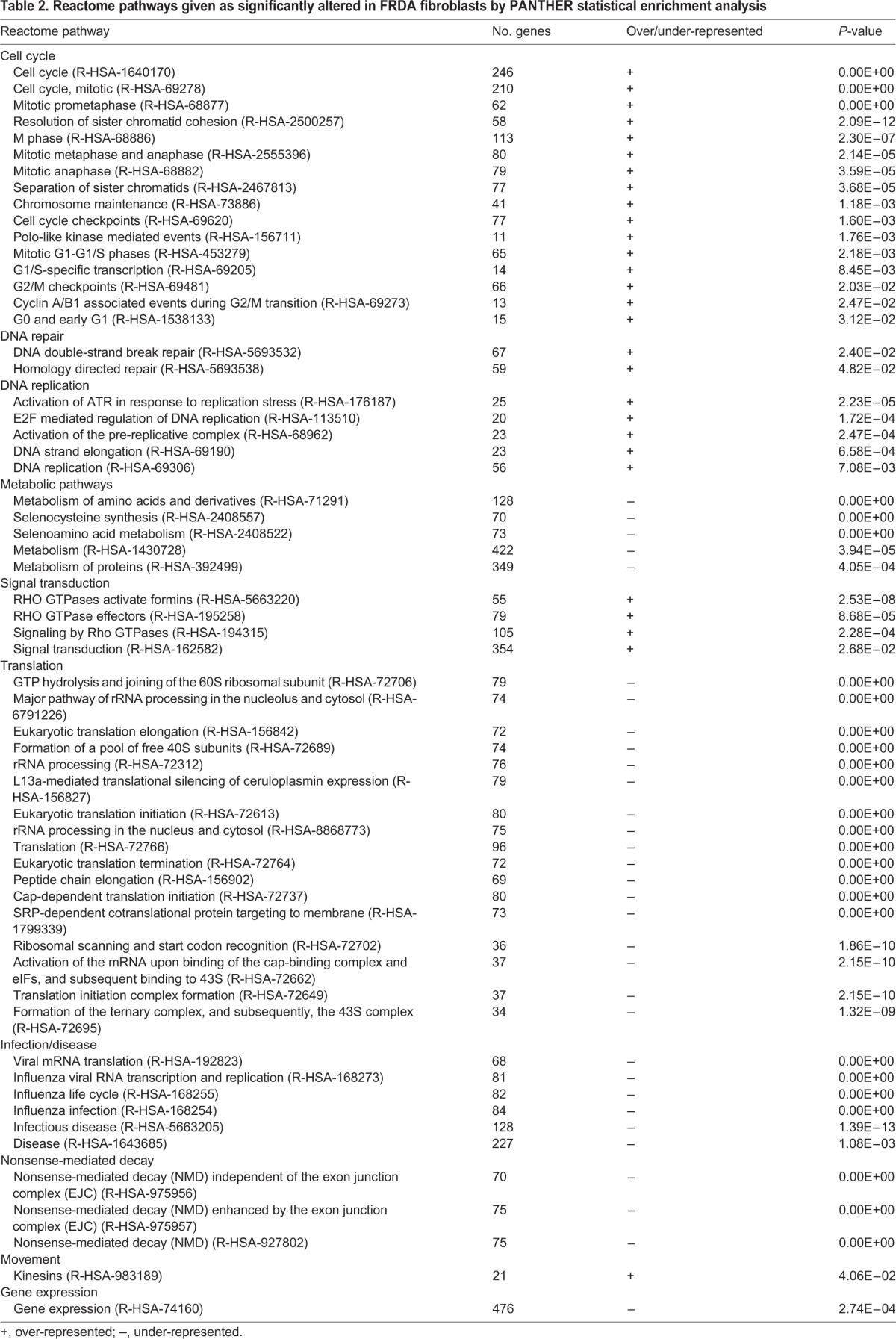
**Reactome pathways given as significantly altered in FRDA fibroblasts by PANTHER statistical enrichment analysis**

### Comparison of expression levels of common normalizer genes between CTRL and FRDA fibroblast sample sets

**Fig. 5.**
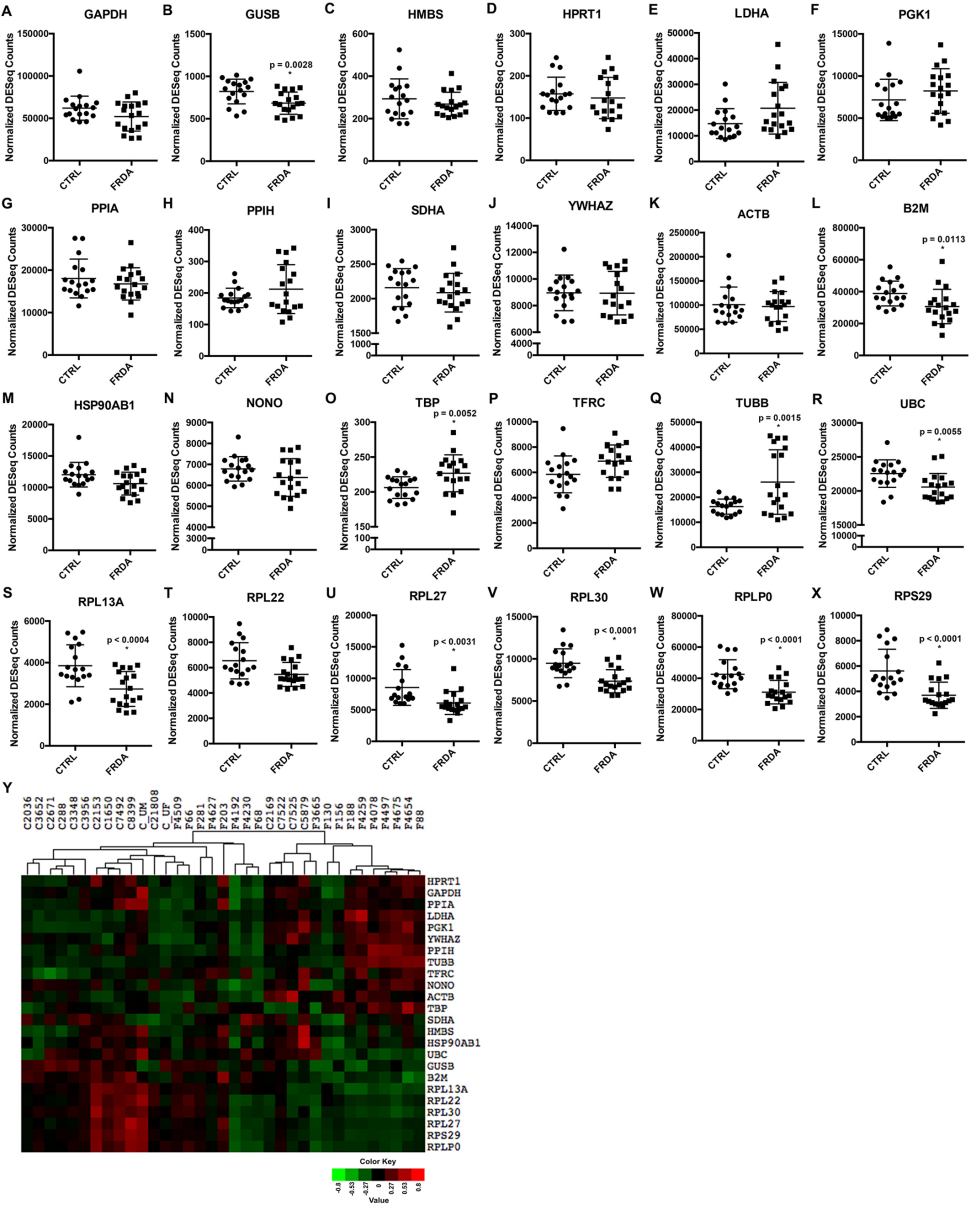
**Assessment of expression levels of common endogenous control genes in CTRL and FRDA fibroblasts.** (A–X) The normalized DESeq counts are plotted for each CTRL and FRDA fibroblast sample for the indicated gene. Outer horizontal bars represent s.d. and the middle bar indicates the mean. Where applicable, statistical significance is denoted by an asterisk and associated *P*-value above the FRDA data points. (Y) The normalized DESeq counts were used to generate expression heatmaps for all 24 normalizer genes. The CTRL and FRDA samples were arranged by hierarchical clustering.

A common obstacle for investigators to overcome for gene and protein expression studies in any field is choosing appropriate normalizer gene(s) when comparing expression levels between sample groups. The large number of CTRL and FRDA fibroblast samples included in this RNA-Seq experiment provides an excellent platform to analyze the expression of normalizer genes in a statistically meaningful way. A panel of 24 endogenous control genes was selected based on literature review (Vandesompele et al., 2002), our previous work (Li et al., 2015c) and those included in The Human Housekeeping Genes RT^2^ Profiler™ PCR Array (Qiagen). These commonly used endogenous control genes encode proteins involved in a wide range of cellular functions, including metabolism and cell structure. The normalized DESeq counts for all samples plotted for each gene indicate that the expression levels of 14 of these commonly used normalizer genes are not statistically different between the CTRL and FRDA fibroblast groups (Fig. 5A,C-K,M-P,T), whereas expression levels of 10 are significantly different between the CTRL and FRDA fibroblast groups (Fig. 5B,L,O,Q-S,U-X). The difference in expression is especially apparent for the included ribosomal genes, for which decreased expression is observed for five out of six of these genes in FRDA samples (Fig. 5S-X; Fig. 3C,D). As expected, a random distribution of CTRL and FRDA fibroblast samples following unsupervised hierarchical clustering is observed, indicating poor separation of these groups based on expression of the selected endogenous control genes (Fig. 5Y). Additionally, geNorm/qbase+ analysis (Vandesompele et al., 2002) was performed to check the stability of the 14 normalizer genes for which expression was not statistically different between the CTRL and FRDA sample groups (Fig. S4). The results show that these genes have high expression stability (average geNorm *M*≤0.5), with *YWHAZ* and *NONO* as the two most stably expressed genes. Taken together, these results indicate that selection of gene expression normalizers should involve the rigorous evaluation of several genes in multiple CTRL and FRDA patient samples.

### Comparison of FRDA gene expression signatures obtained from different peripheral tissues

**Fig. 6.**
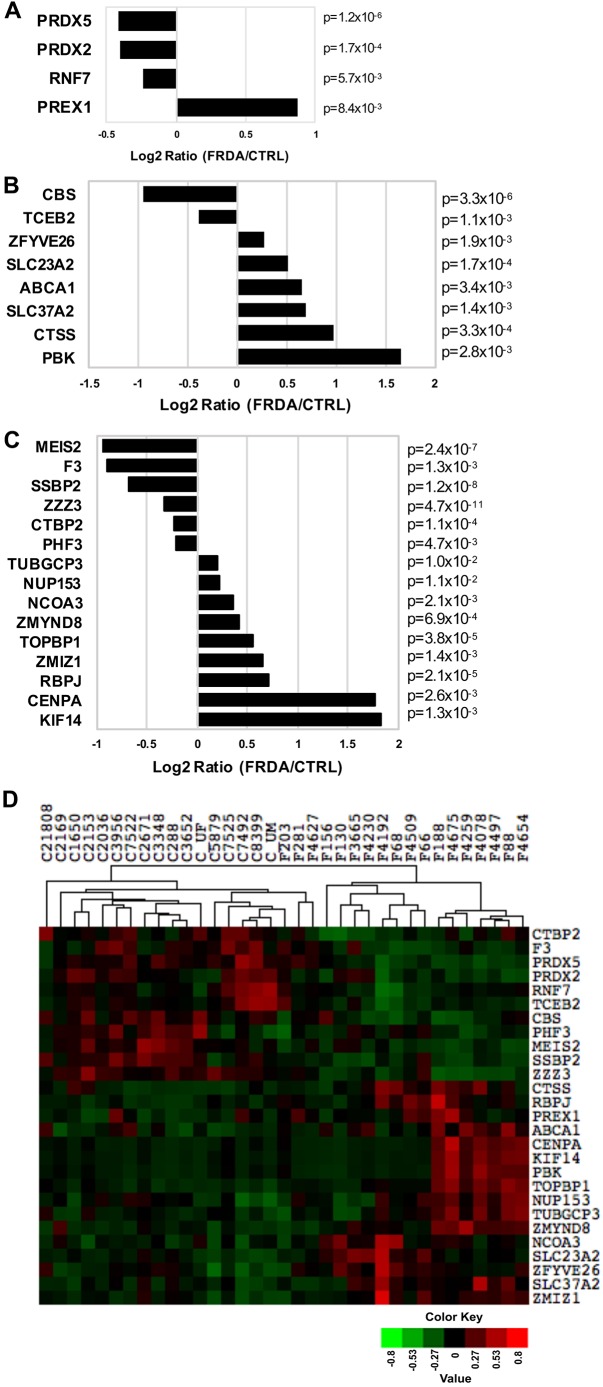
**Overlapping gene expression signatures in FRDA peripheral tissue samples.** The list of 3788 significantly changed genes in fibroblasts was compared to previously published gene expression datasets generated from control and FRDA lymphoblasts or lymphocytes. The bar plots depict the log_2_ ratio (FRDA/CTRL) for gene expression in fibroblast samples with associated *P*-values indicated to the right of each bar. (A) The expression ratios are plotted for four genes found to be significantly changed in both adult FRDA fibroblast and lymphoblast samples (Hayashi and Cortopassi, 2016). (B) The expression ratios of eight genes found to be significantly changed in both adult FRDA fibroblast and PBMC samples are plotted [P77 set (Coppola et al., 2011)]. (C) The expression ratios of 15 genes found to be significantly changed in adult FRDA fibroblast samples and a targeted set of genotoxic stress genes identified as differentially expressed in CTRL and FRDA PBMC samples are plotted (Haugen et al., 2010). (D) The normalized DESeq counts from the fibroblast RNASeq experiment were used to generate an expression heatmap for the 27 significantly changed genes identified in panels A-C. The CTRL and FRDA samples were arranged by hierarchical clustering.

In order to determine whether an FRDA gene expression signature can be defined using samples obtained from different peripheral patient tissues, the 3788 genes differentially expressed between FRDA and CTRL fibroblasts were compared to previously published datasets produced using FRDA and CTRL lymphoblasts or lymphocytes (Coppola et al., 2011; Haugen et al., 2010; Hayashi and Cortopassi, 2016). These studies were selected as examples of targeted (Hayashi and Cortopassi, 2016) and global (Coppola et al., 2011; Haugen et al., 2010) efforts to uncover altered gene expression patterns in FRDA cells. First, the Oxidative Stress and Antioxidant Defense qPCR array (Qiagen) was employed by Hayashi and Cortopassi using eight FRDA and seven CTRL lymphoblast cell lines (Hayashi and Cortopassi, 2016). Out of 84 genes interrogated, 10 were found to be significantly changed in the FRDA cells, namely *PDLIM1*, *EPX*, *GPX2*, *PREX1*, *PRDX5*, *RNF7*, *DUSP1*, *PRDX2*, *NCF2* and *SFTPD* (Hayashi and Cortopassi, 2016). The expression levels of four of these genes, *PRDX5*, *PRDX2*, *RNF7* and *PREX1*, are also significantly changed in FRDA fibroblasts compared to CTRL fibroblasts (Fig. 6A). Although not significantly changed in the lymphoblast screen (Hayashi and Cortopassi, 2016), an additional PRDX family member, *PRDX6*, is also downregulated in FRDA fibroblasts (log_2_ ratio=−0.648, *P*=4.5E–03) (Table S2). Notably, the expression for all four genes is changed in the same direction in both FRDA lymphoblasts and fibroblasts (Hayashi and Cortopassi, 2016). Because decreased expression of *Prdx3* and *Txnrd2* was reported in DRG neurons of the YG8R FRDA mouse model (Shan et al., 2013), we were prompted to analyze our gene list for changes in expression of thioredoxin genes as well. The *TXNRD2* gene is also significantly decreased in FRDA fibroblasts relative to the CTRL cells (log_2_ ratio=−0.299, *P*=8.83E–06) (Table S2), further implicating deficient expression of antioxidant genes in FRDA pathophysiology.

Gene expression patterns in peripheral blood mononuclear cells (PBMCs) isolated from FRDA patients, related FRDA carriers and unrelated control individuals were defined by microarray experiments carried out by Coppola and colleagues (Coppola et al., 2011). A list of 77 differentially changed genes (P77 set) was identified by comparing the gene expression profiles of lymphocytes isolated from 10 pairs of FRDA patients and their related carriers. Changes in expression for the P77 set were also reported between the FRDA patients and 11 unrelated control individuals (Coppola et al., 2011). Eight of the P77 set genes were found to be significantly changed between FRDA and CTRL fibroblasts using unbiased RNA-Seq, namely *CBS*, *TCEB2*, *ZFYVE26*, *SLC23A2*, *ABCA1*, *SLC37A2*, *CTSS* and *PBK* (Fig. 6B, Table S4). The majority of these genes (6/8) were upregulated or downregulated in the same direction for FRDA versus CTRL samples in both studies, whereas *TCEB2* showed slight upregulation in lymphocytes and downregulation in fibroblasts, and *PBK* was downregulated in lymphocytes but upregulated in fibroblasts (Fig. 6B, Table S4).

An independent microarray experiment performed by Haugen et al. again analyzed gene expression changes in PBMCs isolated from adult FRDA patients or unrelated control individuals (Haugen et al., 2010). Through their analysis, Haugen and colleagues found 23 gene sets associated with the genotoxic stress response to be enriched in FRDA samples, and identified 81 genes in common between these sets as a DNA damage signature for FRDA (Haugen et al., 2010). When this list of 81 genotoxic stress genes was compared with the 3788 differentially expressed genes in FRDA fibroblasts, 15 were found to be significantly changed, with eight genes changed in the same direction in lymphocytes and fibroblasts (upregulated: *TOPBP1*, *RBPJ*, *CENPA*, *KIF14*; downregulated: *SSBP2*, *ZZZ3*, *CTBP2*, *PHF3*), whereas two genes were upregulated in lymphocytes but are downregulated in fibroblasts (*MEIS2*, *F3*) and five genes were downregulated in lymphocytes but are upregulated in fibroblasts (*TUBGCP3*, *NUP153*, *NCOA3*, *ZMYND8*, *ZMIZ1*) (Fig. 6C, Table S4). Finally, the normalized DESeq counts for all 27 genes found to be significantly changed in FRDA blood cells and fibroblasts were used to generate a heatmap (Fig. 6D). The CTRL and FRDA fibroblast samples are well separated following unsupervised hierarchical clustering, and the expression levels of most of the genes are uniformly changed among the sample groups. The full names and associated functions for all genes significantly changed in both FRDA fibroblasts and blood cells are given in Table S4.

In addition to the targeted comparisons shown in Fig. 6 and Table S4, we also compared the 3788 differentially expressed genes in FRDA fibroblasts with the uncurated differentially expressed gene lists published for FRDA blood cells in the aforementioned studies (Coppola et al., 2011; Haugen et al., 2010; Hayashi and Cortopassi, 2016) (Table S5). Approximately 19% of the 1283 differentially expressed genes published by Coppola and colleagues were also significantly changed in fibroblasts (Table S5) (Coppola et al., 2011). Likewise, 21% of the 84 genes included in the Oxidative Stress and Antioxidant Defense qPCR array (Qiagen) were significantly altered in FRDA fibroblasts (Table S5) (Hayashi and Cortopassi, 2016). Finally, approximately 14% of the 2874 differentially expressed genes in adult FRDA PBMC samples reported by Haugen et al. were also significantly dysregulated in FRDA fibroblast cells (Table S5) (Haugen et al., 2010). Taken together, these data suggest that peripheral tissues can provide a reasonable platform for defining universal changes in gene expression related to FRDA.

## DISCUSSION

Conducting -omics studies with a statistically relevant number of samples, especially for rare diseases, is challenging. Gathering larger numbers of specimens and/or uniformly established cell lines requires a significant amount of effort. One important point that this study highlights is the need for conducting expression profiling or biomarker discovery screens on an FRDA cohort of appropriate sample size. For example, from the statistical enrichment test results it appears that there is a general upregulation of genes involved in DNA replication and DNA repair in FRDA samples compared to CTRL (Fig. S3B), when in fact the result is skewed by an extreme upregulation of genes in a subset of seven FRDA patients that is not seen throughout the entire group of 18 (Fig. S3D,F). Although this result might reveal a true biological insight with regards to heterogeneity within the FRDA patient population, it also highlights the need for caution when interpreting data from studies performed with a small sampling size. Nevertheless, the unbiased data produced from this RNA-Seq experiment can be used to generate hypotheses for pilot studies. Moreover, the parallels drawn between our fibroblast RNA-Seq dataset and gene expression changes or phenotypes published from disease-relevant tissues (e.g. nervous system and heart tissues) can also be used as a starting point for validation studies in FRDA-affected cell types. Although all of the significant results reported herein deserve attention, we will focus our discussion to a few novel findings that we feel are particularly relevant and of general interest to the field.

GO analyses of the RNA-Seq data revealed that the broadly defined category of ‘translation’ is enriched when comparing transcription signatures of the FRDA and CTRL cohorts, indicating that protein synthesis may be impaired in FRDA cells. Although GAA expansions impede transcription of the *FXN* gene, leading to frataxin protein deficiency, metabolic changes evoked by low frataxin levels might decrease the efficiency of other cellular processes including translation or even transcription itself. Enrichment of protein biosynthesis categories in our GO analyses supports this hypothesis, and has been reported by others as well (Haugen et al., 2010).

Further examination of genes belonging to the translation GO term revealed a widespread decrease in expression of several cytoplasmic (Fig. 3E) and mitochondrial (Fig. 3F) aaRS genes. These enzymes are responsible for specific attachment of amino acids with appropriate tRNAs, a critical step of protein synthesis. Interestingly, mutations in aaRS genes and their nuclear-encoded mitochondrial counterparts (aaRS2s) have been linked to several inherited disorders. Frequently, clinical presentation of these diseases includes phenotypic characteristics of mitochondrial disorders, including neurological and cardiac symptoms observed also in FRDA patients. Mutations in *MARS* have been linked to Charcot-Marie-Tooth (CMT) type 2U (OMIM #616280), with affected individuals experiencing peripheral neuropathy characterized by progressive muscle weakness and atrophy, thus resembling some aspects of FRDA. Similarly, heterozygous mutations in *AARS* were identified in cases of CMT type 2N (CMT2N; OMIM #613287). Recently, mutations in *QARS* were identified as causative variants of a complex neuron-developmental phenotype (Kodera et al., 2015; Zhang et al., 2014) that included atrophy of the cerebellum, which is frequently found in FRDA autopsy and MRI studies (Koeppen et al., 2012; Solbach et al., 2014). Although the *TARS* gene has not yet been definitively linked with any genetic disorders, we found its expression to be significantly downregulated in FRDA fibroblasts (Fig. 3E) as well as lymphocytes (Coppola et al., 2011; Haugen et al., 2010).

In addition, mutations in genes encoding the mitochondrial aaRS2 enzymes found to be downregulated in FRDA cells have been recurrently linked to various neurological pathologies (reviewed in Diodato et al., 2014). Mutations in the *NARS2* gene, downregulated in the FRDA cohort, are known to cause a spectrum of clinical phenotypes, including non-syndromic hearing loss, myopathy, and the mitochondrial energy deficiency disorders Leigh syndrome and Alpers syndrome (Mizuguchi et al., 2017; Simon et al., 2015; Sofou et al., 2015). Furthermore, along with mutations that affect NARS2 enzymatic function, decreased levels of *NARS2* mRNA and protein are observed in fibroblasts from individuals with Leigh syndrome (Simon et al., 2015). Homozygous mutations in *NARS2* also can lead to combined oxidative phosphorylation deficiency 24 (COXPD24; OMIM #616239), whereas heterozygous mutations in *VARS2* results in a similar condition termed combined oxidative phosphorylation deficiency 20 (COXPD20; OMIM #615917) (Simon et al., 2015). Both conditions are characterized molecularly by defective mitochondrial respiration and clinically with ataxia, amongst other neuromuscular symptoms.

Because expression of several aaRS and aaRS2 genes is decreased in FRDA cells, a cumulative effect of their deficiency, either directly related to the pool of aa-tRNAs available or other as-yet uncovered functions of these enzymes, can affect the clinical presentation of FRDA patients. Moreover, individual differences in expression levels of selected aaRS genes can modulate predisposition to cardiomyopathy, hearing loss or other FRDA symptoms. Further investigation of the expression and function of aaRS and aaRS2 enzymes in FRDA-disease-relevant models, such as neurons derived from induced pluripotent stem cells (iPSCs) and mouse models, is necessary to elucidate their potential role in pathogenesis.

Currently, there are 52 annotated SLC transporter families, encompassing 395 genes, in mammals (Hediger et al., 2013). These genes include passive and active membrane transporters that move solutes (Na^+^, Ca^2+^, PO_4_^3−^, etc.) and small molecules (amino acids, monoamines, sugars, etc.) within and between cells to maintain cell physiology and evoke action when signals arise. Many SLC transporters are targets of therapeutic intervention for neurological and cardiovascular diseases (César-Razquin et al., 2015; Hediger et al., 2013; Rask-Andersen et al., 2013), although they have not yet been a focus of investigation for FRDA.

The expression of eight genes belonging to the *SLC25* family are significantly changed in FRDA fibroblasts, including the most significantly changed SLC gene in our analysis, *SLC25A3*. Deleterious mutations in SLC25A3 are implicated in mitochondrial phosphate-carrier deficiency disorder (OMIM #610773), which, like FRDA, features cardiomyopathy as a major symptom (Mayr et al., 2007). Likewise, the most downregulated SLC gene in the FRDA fibroblasts is the *SLC8A1* gene (also known as *NCX1*), which encodes a Na^+^/Ca^2+^ exchanger required for heart muscle contractions (Komuro et al., 1992; Ebert et al., 2005). These results suggest that altered intra-cellular (mitochondrial) and inter-cellular solute transport might play a role in the molecular physiology underlying the cardiac symptoms observed in some FRDA patients, and that further evaluation and validation of SLC expression in disease-relevant models, such as iPSC-differentiated cardiomyocytes, are warranted.

Members of the SLC6 gene family are plasma membrane transporters that primarily shuttle amino acids, neurotransmitters or osmolytes between the extracellular space and various cells within the CNS (Kristensen et al., 2011). The most highly upregulated SLC gene in the FRDA fibroblasts is *SLC6A7*, which encodes an L-proline transporter thought to regulate excitatory presynaptic transmission (Shafqat et al., 1995; Velaz-Faircloth et al., 1995). Other SLC6 gene family members upregulated in the FRDA samples include those encoding a Na^+^/Cl^−^-dependent serotonin transporter (*SLC6A4*) localized in the plasma membranes of cells in CNS tissues as well as in neurons of the peripheral nervous system (Ramamoorthy et al., 1993), and a Na^+^-dependent branched-chain-amino-acid transporter (*SLC6A15*) predominantly expressed in the brain (Takanaga et al., 2005). Given that the expression of many SLC transporters is tissue specific, the detectable changes of these genes in FRDA fibroblasts suggests that their dysregulation could be a general underlying disease feature that affects all cells.

Our comparison of differentially expressed genes in FRDA fibroblasts with expression signatures previously published using FRDA lymphocytes revealed several common classes of dysregulated genes. First, downregulation of genes involved in the oxidative stress response was observed, including the peroxiredoxin genes *PRDX2*, *PRDX5* and *PRDX6*. These enzymes protect cells from super-physiological levels of hydrogen peroxide, alkyl hydroperoxides and peroxynitrite by participating in coupled redox reactions with thioredoxin enzymes to reduce these damaging molecules (McBean et al., 2015). PRDX6 additionally functions in the repair of peroxidized membrane phospholipids (Li et al., 2015a) and in clearance of damaged mitochondria (Ma et al., 2016). Evidence for increased levels of reactive oxygen species and resulting detrimental consequences (e.g. accumulation of protein and lipid adducts, membrane damage, etc.) has been collected throughout the years from various FRDA eukaryotic model systems (Abeti et al., 2016; Babcock et al., 1997; Busi et al., 2006; Cavadini et al., 2000; Chantrel-Groussard et al., 2001; Hayashi and Cortopassi, 2016; Napoli et al., 2006; Shan et al., 2013; Wong et al., 1999). Therefore, these results are not unexpected and further support the notion that deficient expression of a number of antioxidant defense genes persistently observed in FRDA models and patient cells may contribute to their increased sensitivity to oxidative stress.

In the Reactome Pathway database, selenocysteine synthesis (R-HAS-2408557) and selenoamino acid metabolism (R-HAS-2408522) are among the most significantly downregulated pathways associated with the 3788 differentially expressed genes in FRDA fibroblasts (Table 2). Consistent with previous findings, we observe statistically significant decreases in expression of *SARS* (Fig. 3E), *SHMT2* (log_2_ ratio=−0.445, *P*=0.001) and *TST* (log_2_ ratio=−0.344, *P*=0.007) in FRDA fibroblasts (Tan et al., 2003). Moreover, the gene encoding cystathione beta synthase (*CBS*), the enzyme responsible for the first step in the transsulfuration pathway, is decreased in our dataset (log_2_ ratio=−0.955, *P*=3.29E–06, FDR<0.001) (Fig. 6B, Table S2), as well as in FRDA lymphocytes (Fig. 6B) (Coppola et al., 2011). Furthermore, significantly decreased expression of *CBS* was reported in the adult FRDA lymphocyte differentially expressed gene list (2874 genes) published by Haugen and colleagues (Table S5) (Haugen et al., 2010). The consistent and robust decrease in expression of this enzyme in FRDA peripheral tissues as detected by independent platforms indicates *CBS* expression as a potential biomarker.

The GO analyses of data obtained from our unbiased RNA-Seq platform revealed enrichment of processes consistently associated with FRDA pathogenesis, such as DNA repair and sulfur amino acid metabolism, while highlighting novel areas for FRDA research, such as regulation of membrane solute carriers or aaRS activity. Although our gene expression dataset represents differences observed between CTRL and FRDA fibroblasts grown under optimal glycolytic conditions, the possibility exists that unique and robust gene expression signatures could be evoked by culturing cells in a setting that requires mitochondrial function for survival, for instance with an alternative energy source (e.g. galactose). Certainly, gene expression studies performed on samples cultured under various metabolic conditions or different drug treatment regimens are of interest, and such transcriptome data could be compared to the gene expression signature of FRDA cells obtained under routine culture conditions presented herein as a reference. The large sample size used in our experiment combined with the high sensitivity of RNA-Seq allowed us to detect subtle yet significant changes in gene expression in FRDA samples that might otherwise go undetected if one of these parameters is forgone. Taken together, the gene expression patterns defined for FRDA fibroblasts reported herein strengthen the rationale for including these cells as models for molecular mechanism and gene expression biomarker studies.

## MATERIALS AND METHODS

### Primary fibroblast isolation and culture

Skin biopsy samples were obtained and all studies were conducted in accordance with approvals of Children's Hospital of Philadelphia (CHOP) and University of Alabama (UAB) Institutional Review Boards (CHOP IRB #10-007864; UAB IRB #N131204003) (Li et al., 2016). Primary fibroblast lines were established from punch skin biopsy samples as described in detail by Li et al. (2015b, 2016). Once established, primary fibroblast cultures were maintained in DMEM high glucose (Life Technologies, Carlsbad, CA) containing 15% fetal bovine serum (Hyclone) and 1% non-essential amino acids (Life Technologies, Carlsbad, CA). Cells were passaged by incubating in 0.25% trypsin-EDTA (Life Technologies) for 5 min and harvested for RNA isolation when they reached ∼80-85% confluence.

### RNA isolation, sample preparation and RNA-Seq

The complete protocols for RNA isolation, library preparation and RNA-Seq reaction preparation were previously published (Li et al., 2015b). Cells were harvested by trypsinization, centrifuged and rinsed once with ice-cold phosphate-buffered saline (PBS). Total RNA was isolated using a Qiagen RNeasy Mini Kit (Qiagen, Valencia, CA) followed by an independent DNase I treatment using the TURBO DNA*-free* system according to the manufacturer's recommended rigorous protocol (Ambion, Carlsbad, CA). RNAs isolated from all 35 fibroblast lines (18 FRDA and 17 CTRL) (1 µg per sample) were reverse transcribed to generate sequencing libraries using Truseq Stranded Total RNA Library Prep kit (Illumina) and sequenced by HiSeq2000 (Illumina) at the University of Texas MD Anderson Cancer Center Molecular Biology core facility within the Department of Epigenetics and Molecular Carcinogenesis at the Virginia Harris Cockrell Cancer Research Center, Science Park (Li et al., 2015b). Approximately 27- to 39-million sequencing reads were generated for each fibroblast mRNA preparation and 81-94% of fragments were mapped by both ends to the human genome (hg19) using TopHat (version 2.0.7) (Kim et al., 2013) and bowtie2 (version 2.1.0) (Langmead and Salzberg, 2012).

### Differential gene expression analyses

As previously published (Li et al., 2015b), the number of fragments in each gene annotated in the RefSeq database (Pruitt et al., 2014) was numbered using htseq-count from the HTSeq package (version 0.5.3p9, HTSeq: https://pypi.python.org/pypi/HTSeq/0.5.3p7). Only genes with more than 10 fragments in all the samples were included in differential expression analysis. Differential gene expression between CTRL and FRDA fibroblast samples was statistically assessed using the R/Bioconductor DESeq package (version 1.10.1) (Anders and Huber, 2010). An FDR≤0.05 served as the cutoff for differential expression between the two groups. The normalized DESeq counts for each sample were used to calculate average log_2_ expression values for the CTRL and FRDA groups. The log_2_ ratio values for each gene were then calculated as the average log_2_ expression value for the FRDA sample group subtracted by the average log_2_ expression value for the CTRL sample group. geNorm analysis (Vandesompele et al., 2002) was performed on the 14 normalizer genes in qbase+ (version 3.1) using log_2_-normalized counts by DESeq.

### Hierarchical clustering and heatmaps

Gene expression heatmaps were generated using the Cluster 3.0 and Java Tree View applications. Briefly, gene lists and associated normalized DESeq counts were uploaded as input files. The DESeq counts of each gene across samples were centered by mean and normalized before clustering. Then the genes were clustered based on Euclidean distance and average linkage clustering method. The samples were clustered by centered correlation and average linkage clustering method.

### GO, statistical over-representation and statistical enrichment analyses

The PANTHER online resource (www.pantherdb.org) was used for all GO tests, statistical over-representation tests and statistical enrichment tests (Mi et al., 2013, 2016, 2017). The statistical over-representation tests were performed using default settings after entering the list of 3788 differentially expressed genes as an ID list with *Homo sapiens* selected as the organism database to search (analysis type: PANTHER Over-representation Test, released 07/15/2016; annotation version and release date: PANTHER version 11.1, released 10/24/2016). For results presented herein, a Bonferroni correction was applied for each test.

The statistical enrichment tests were performed using default settings after uploading a tab-delimited text file containing the 3788 differentially expressed genes and their associated log_2_ ratio (FRDA/CTRL) values (analysis type: PANTHER Enrichment Test, released 10/20/2016; annotation version and release date: PANTHER version 11.1, released 10/24/2016). For results presented herein, a Bonferroni correction was applied for each test. The 3788 differentially expressed gene list was used as input for the Enrichr pipeline (Chen et al., 2013; Kuleshov et al., 2016). Results from KEGG 2016 and WikiPathways 2016 were exported in table format and are presented as Table S3.

## Supplementary Material

Supplementary information
