# Supplementary material for: Comprehensive analysis of gene expression patterns in Friedreich's ataxia fibroblasts by RNA sequencing reveals altered levels of protein synthesis factors and solute carriers
Source: Dis Model Mech. 2017 Nov 1;10(11):1353–69. doi: 10.1242/dmm.030536 (PMC5719256; doi:10.1242/dmm.030536)
Supplement: Supplementary information [file dmm-10-030536-s1.pdf]

Fig. S1, Napierala et al.

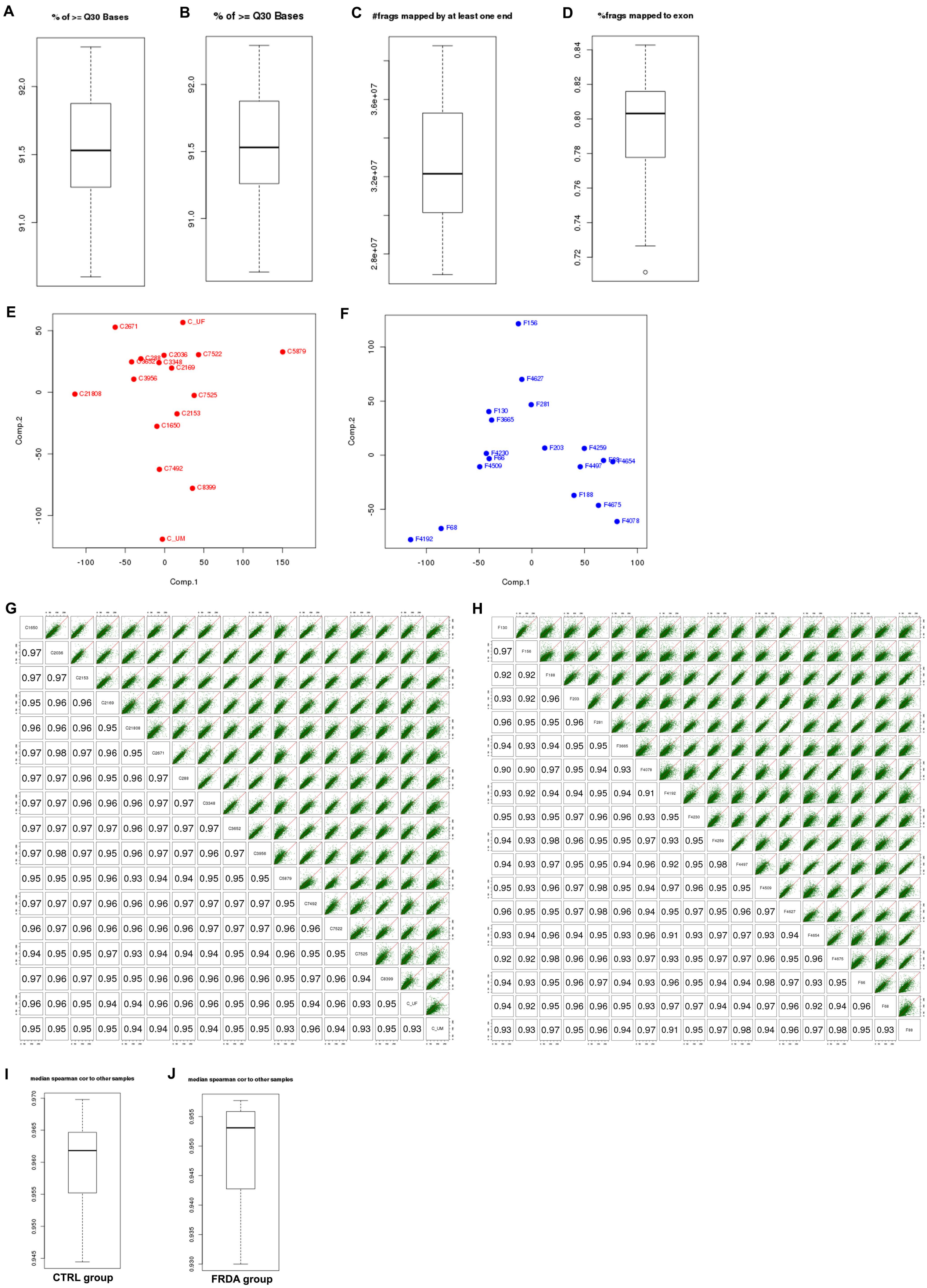

Disease Models & Mechanisms • Supplementary information

Fig. S1. Determination of intra-group heterogeneity and outlier analysis. First, it was determined if any of the samples is an outlier in terms of basic quality, such as % of bases with quality score ≥ 30 (A), mean quality score (B), number of fragments mapped (C), and % of fragments mapped to exons (D). No samples were determined to be outliers (defined as outside 1.5 times the interquartile range above the upper quartile and bellow the lower quartile), except one sample with relatively lower % of fragments mapped to exons (D). As the % of fragments mapped to exons for this sample is still as high as 71%, this sample was considered acceptable and was not removed from the analysis. Next, PCA plots were generated for the samples in each group separately (E, F). By visual inspection, no outliers were identified. To statistically evaluate if any of the sample is an outlier within groups, the median spearman distance of each sample to rest of the samples in the same group was calculated (G, H) and boxplots were generated for the two groups (I, J). Again, no outliers were identified within either group.

Fig. S2, Napierala et al.

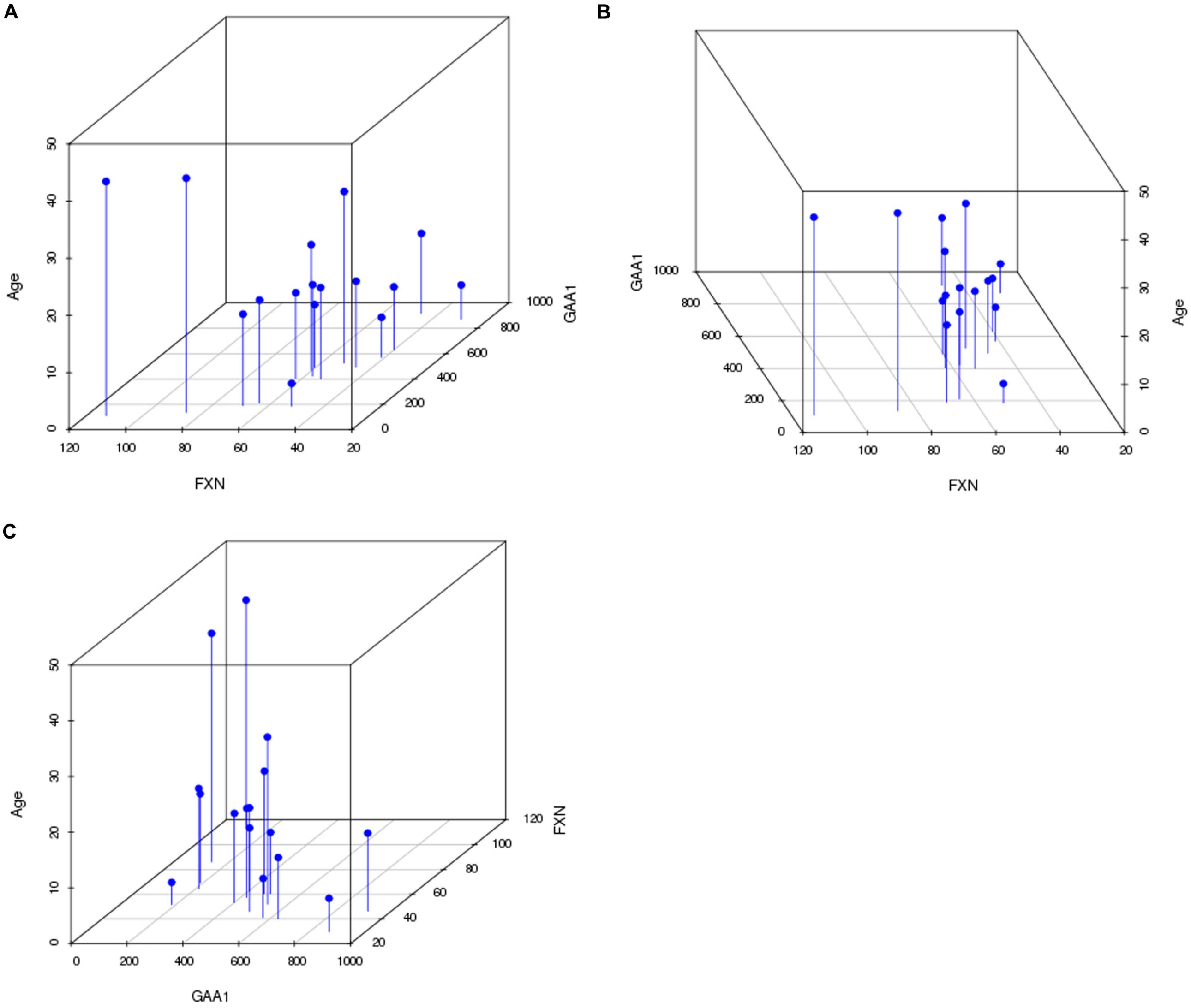

Fig. S2. Correlation of GAA repeat length, FXN expression, and age of onset as determined from FRDA fibroblasts. Shown are three-dimensional plots of GAA1 repeat length on the x-axis (GAA1), age of disease onset on the y-axis (age), and *FXN* expression by RNA sequencing on the z-axis (FXN) for each FRDA fibroblast sample. The viewing angles shown are (A) 220°, (B) 300°, and (C) 40°.

Fig. S3, Napierala et al.

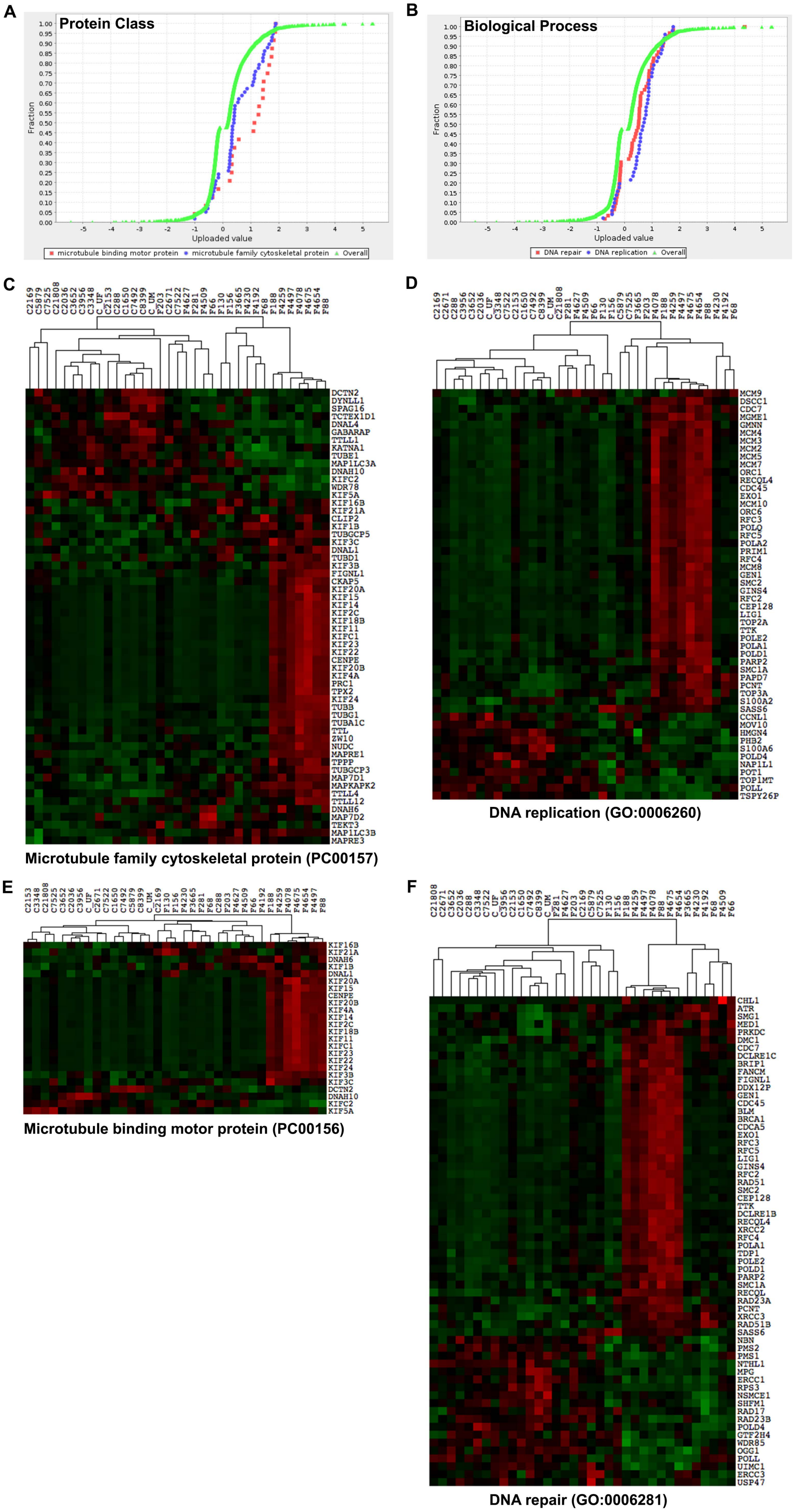

Disease Models & Mechanisms • Supplementary information

Fig. S3. Statistical enrichment analysis for significantly changed genes in FRDA fibroblasts. Plots were generated from the PANTHER statistical enrichment test tool using the list of 3,788 significantly changed genes and their associated log2 ratio values. (A) Genes mapped to the PANTHER Protein Class categories microtubule binding motor protein (PC00156) and microtubule family cytoskeletal protein (PC00157) are plotted in red and blue, respectively, with the overall distribution of expression for the 3,788 genes shown in green. (B) Genes mapped to the PANTHER GO-Slim Biological Process categories DNA repair (GO: 0006281) and DNA replication (GO: 0006260) are plotted in red and blue, respectively, with the overall distribution of expression for the 3,788 genes shown in green. (C) The normalized DESeq counts were used to generate an expression heatmap for 58 genes mapped to microtubule family cytoskeletal protein (PC00157). The CTRL and FRDA fibroblast samples are arranged hierarchically. (D) The normalized DESeq counts were used to generate an expression heatmap for 52 genes mapped to DNA replication (GO: 0006260). The CTRL and FRDA fibroblast samples are arranged hierarchically. (E) The normalized DESeq counts were used to generate an expression heatmap for 24 genes mapped to microtubule binding motor protein (PC00156). The CTRL and FRDA fibroblast samples are arranged hierarchically. (F) The normalized DESeq counts were used to generate an expression heatmap for 62 genes mapped to DNA repair (GO: 0006281). The CTRL and FRDA fibroblast samples are arranged hierarchically.

Disease Models & Mechanisms • Supplementary information

Fig. S4, Napierala et al.

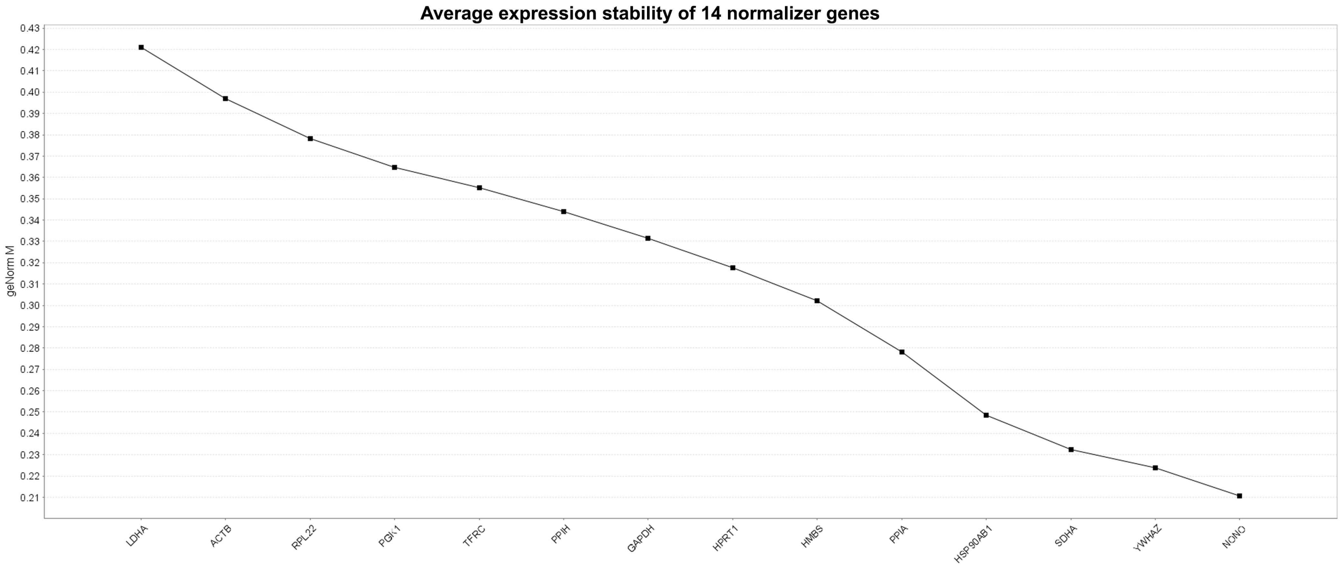

Fig. S4. GeNorm/qbase+ analysis of normalizer candidates for FRDA gene expression studies. A GeNorm M plot is shown with the Y axis indicating the average expression stability of the 14 normalizer genes that are not statistically different between CTRL and FRDA fibroblasts. A greater stability in expression receives a lower M value.

Table S1. Molecular and clinical data for FRDA and CTRL fibroblast samples used for RNA Sequencing.

Cell Line	GAA repeat lengths (allele 1, allele 2)	Age of disease onset (years)	Gender	Age at Sampling (years)	Symptoms			*FXN* Normalized DESeq Counts
					C	D	H	
**FRDA**								
F281	630, 806	11	M	19	+	-	+	40.08343355
F4627	468, 807	22	F	50	-	-	-	60.30978005
F203	916, 1382	14	F	31	-	-	-	46.26690129
F130	136, 540	41	M	56	+	-	-	86.08670109
F156	495, 505	15	M	41	-	-	-	45.9628706
F3665[1]	816, 1410	ND	F	13	ND	ND	ND	39.50923754
F4230	870, 1470	6	F	28	+	+	+	29.61076192
F4192	400, 967	16	M	33	-	-	-	53.22397838
F68	570, 1200	7	F	21	+	-	+	41.14274262
F4509	211, 1428	18	M	36	-	-	-	64.32614921
F66	110, 590	41	M	70	-	-	-	112.9722268
F188	490, 680	11	M	47	-	-	+	60.31816612
F4259	404, 920	15	M	37	+	-	+	62.23261322
F4497	526, 826	30	F	44	-	-	+	51.87681863
F4078[1]	341, 480	ND	M	30	ND	ND	ND	94.71514654
F4654	190, 500	16	M	19	+	-	+	68.99572404
F88	422, 520	16	F	50	-	-	+	57.26501829
F4675	185, 1130	4	M	28	-	-	-	51.50945247
**Mean**	**GAA1=454, GAA2=898**	**17.69**		**36.28**				**59.24**
**StdDev**				**14.73**				**20.95**
**Coefficient of Variation (CV)**				**40.6**				**35.36**
**T-test**								**4.21E-12**
**CTRL**								
C21808[1]	N/A	N/A	M	1 day	N/A	N/A	N/A	161.2991848
C2671[1]	N/A	N/A	F	47	N/A	N/A	N/A	98.08969462
C2036[1]	N/A	N/A	F	11	N/A	N/A	N/A	123.2468382
C288[1]	N/A	N/A	M	64	N/A	N/A	N/A	138.5481289

Disease Models & Mechanisms • Supplementary information

C3348[1]	N/A	N/A	M	10	N/A	N/A	N/A	121.0665267
C3652[1]	N/A	N/A	M	24	N/A	N/A	N/A	124.1303539
C3956[1]	N/A	N/A	F	27	N/A	N/A	N/A	149.7992743
C_UF	N/A	N/A	F	35	N/A	N/A	N/A	147.1456236
C7522[1]	N/A	N/A	F	19	N/A	N/A	N/A	147.5246792
C2153[1]	N/A	N/A	M	40	N/A	N/A	N/A	166.8292707
C1650[1]	N/A	N/A	F	37	N/A	N/A	N/A	118.7702431
C7492[1]	N/A	N/A	M	17	N/A	N/A	N/A	121.9659664
C8399[1]	N/A	N/A	F	19	N/A	N/A	N/A	125.6609057
C_UM	N/A	N/A	M	39	N/A	N/A	N/A	178.3088782
C2169[1]	N/A	N/A	M	52	N/A	N/A	N/A	102.1403775
C7525[1]	N/A	N/A	F	22	N/A	N/A	N/A	133.8142106
C5879[1]	N/A	N/A	F	48	N/A	N/A	N/A	145.6948052
**Mean**				**31.94**				**135.53**
**StdDev**				**15.77**				**21.85**
**Coefficient of Variation (CV)**				**49.37**				**16.12**

ND - not determined
[1] - denotes fibroblast cell line obtained from Coriell Cell Repositories

Table S2. List of differentially expressed genes (FDR<0.05) and log2 ratio expression values as determined by RNA sequencing for FRDA and CTRL fibroblast samples.

Click here to Download Table S2

Table S3. Pathways that are significantly enriched as revealed by the KEGG 2016 and WikiPathways 2016 databases when the list of differentially expressed genes in fibroblasts (FDR<0.05) is used as input in the Enrichr platform (Chen et al., 2013; Kuleshov et al., 2016).

Click here to Download Table S3

Disease Models & Mechanisms • Supplementary information

Table S4. Genes that were found to be differentially expressed in both FRDA fibroblasts and blood cells.

Symbol	Name	Function	Agreement between datasets
**Hayashi, et al. (2016) *PLoS One* 11.**			
PRDX2	Peroxiredoxin 2	Antioxidant	Yes
PRDX5	Peroxiredoxin 5	Antioxidant	Yes
RNF7	Ring Finger Protein 7	Ubiquitination	Yes
PREX1	Phosphatidylinositol-3,4,5-Trisphosphate Dependent Rac Exchange Factor 1	Guanine nucleotide exchange factor	Yes
**Coppola, et al. (2011) *Ann Neurol* 70**: 790-804.			
CBS	Cystathionine-Beta-Synthase	Transsulfuration reactions	Yes
TCEB2	Transcription Elongation Factor B Subunit 2	Transcription elongation	No
ZFYVE26	Zinc Finger FYVE-Type Containing 26	Transcription regulation	Yes
SLC23A2	Solute Carrier Family 23 Member 2	Tissue-specific Vitamin C uptake	Yes
ABCA1	ATP Binding Cassette Subfamily A Member 1	Membrane cholesterol transporter	Yes
SLC37A2	Solute Carrier Family 37 Member 2	Inorganic phosphate and glucose-6-phospate antiporter	Yes
CTSS	Cathepsin S	Cysteine protease	Yes
PBK	PDZ Binding Kinase	Serine/threonine protein kinase	No
**Haugen, et al. (2010) *PLoS Genet* 6.**			
MEIS2	Meis Homeobox 2	Transcription regulator	No adults; Yes children
F3	Coagulation Factor III, Tissue Factor	Blood coagulation	No
SSBP2	Single Stranded DNA Binding Protein 2	Genome stability maintenance	Yes
ZZZ3	Zinc Finger ZZ-Type Containing 3	Chromatin regulation	Yes
CTBP2	C-Terminal Binding Protein 2	Isoform 1: Transcription regulation<br>Isoform 2: Synapse regulation	Yes
PHF3	PHD Finger Protein 3	Transcription regulation	Yes
TUBGCP3	Tubulin Gamma Complex Associated Protein 3	Microtubule organization at the centrosome	No
NUP153	Nucleoporin 153	Nucleocytoplasmic transport	No
NCOA3	Nuclear Receptor Coactivator 3	Transcription activation and histone acetylation	No adults; Yes children
ZMYND8	Zinc Finger MYND-Type Containing 8	Receptor activity	No
TOPBP1	Topoisomerase (DNA) II Binding Protein 1	DNA replication	Yes
ZMIZ1	Zinc Finger MIZ-Type Containing 1	Transcription regulation	No
RBPJ	Recombination Signal Binding Protein For Immunoglobulin Kappa J Region	Transcription regulation	Yes
CENPA	Centromere Protein A	Centromere chromatin structure	Yes
KIF14	Kinesin Family Member 14	Microtubule motor activity	Yes

Table S5. Lists of significantly changed genes and log2 ratio expression values for those overlapping between the fibroblast dataset (FDR<0.05) and datasets published in (Coppola et al., 2011; Haugen et al., 2010; Hayashi and Cortopassi, 2016).

Click here to Download Table S5
